# Herpes simplex virus type 1 R-loops are targets for APOBEC-mediated mutagenesis

**DOI:** 10.1186/s13059-026-04078-y

**Published:** 2026-04-14

**Authors:** Márton Miskei, Dóra Varga, Lilla Hornyák, Éva Sipos, Éva Nagy, Qiuzhen Li, Zsolt Karányi, Zoltán Szabó, Rachel DeWeerd, Abby M. Green, Dávid Szüts, Eszter Csoma, Lóránt Székvölgyi

**Affiliations:** 1 MTA-DE Momentum Genome Architecture and Recombination Research Group, Department of Molecular and Nanopharmaceutics, Faculty of Pharmacy, University of Debrecen, Debrecen, Hungary; 2 Doctoral School of Pharmaceutical Sciences, Faculty of Pharmacy, University of Debrecen, Debrecen, Hungary; 3 Department of Pharmacodynamics, Faculty of Pharmacy, University of Debrecen, Debrecen, Hungary; 4 Department of Internal Medicine, Division of Endocrinology, Faculty of Medicine, University of Debrecen, Debrecen, Hungary; 5 Department of Emergency Medicine, Faculty of Medicine, University of Debrecen, Debrecen, Hungary; 6 Department of Pediatrics, Center for Genome Integrity, Siteman Cancer Center, Washington University School of Medicine, St. Louis, MO USA; 7 Institute of Molecular Life Sciences, HUN-REN Research Centre for Natural Sciences, Budapest, Hungary; 8 Department of Medical Microbiology, Faculty of Medicine, University of Debrecen, Debrecen, Hungary; 9 Department of Molecular Biotechnology and Microbiology, Faculty of Science and Technology, University of Debrecen, Debrecen, Hungary; 10 HUN-REN-UD Fungal Stress Biology Research Group, University of Debrecen, Debrecen, Hungary

**Keywords:** R-loop, RNA–DNA hybrid, DRIP-seq, APOBEC, Virus, HSV-1

## Abstract

APOBEC3 enzymes are key effectors of antiviral immunity that introduce mutations into viral genomes. We show that viral R-loops serve as preferred substrates for APOBEC3-mediated mutagenesis during herpes simplex virus type 1 (HSV-1) infection. APOBEC3 enzymes are recruited to viral R-loops, generating clustered C-to-T mutations in genes critical for viral assembly. Importantly, R-loops alone are not mutagenic, and APOBEC3 enzymes do not edit HSV-1 without an R-loop; mutagenesis occurs when R-loops and APOBEC3 enzymes interact. These findings identify endogenous R-loops formed during the HSV-1 life cycle as focal vulnerabilities and highlight R-loop–APOBEC3 coupling as a mechanism for antiviral mutagenesis.

**Supplementary Information:**

The online version contains supplementary material available at 10.1186/s13059-026-04078-y.

## Background

APOBEC cytidine deaminases are key enzymes that introduce C-to-T mutations in viral genomes. While best known for their anti-retroviral activity against HIV-1, APOBEC3 (A3) proteins also restrict double-stranded DNA viruses, including herpesviruses [1–5]. Herpes simplex virus type 1 (HSV-1) is a significant human pathogen with a complex dsDNA genome [6, 7]. Despite the known anti-HSV-1 activity of A3 proteins, the efficiency and genomic distribution of APOBEC-mediated mutations remain poorly understood [8–10].

Special nucleic acid structures called R-loops [11–13] have recently been implicated as substrates for certain APOBEC enzymes (particularly A3B) in oncology studies, though the evidence is often correlative [14, 15]. Although herpesviruses are known to harbor R-loop-forming sequences [16–23], it has not been demonstrated whether APOBEC enzymes directly deaminate viral R-loop sites during infection. Here, we show that R-loops serve as specific targets for A3 enzymes within the HSV-1 genome. This finding reveals a previously uncharacterized mechanism driving localized mutagenesis during viral infection and highlights the role of R-loop structures in host defense.

## Results and discussion

**Fig. 1:**
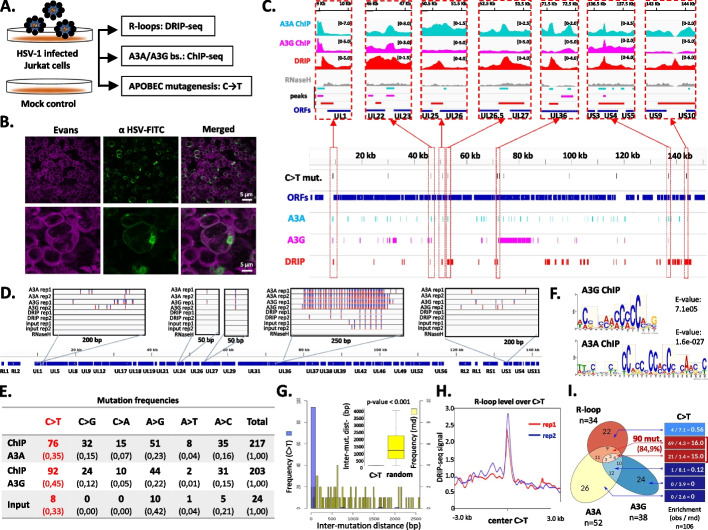
Investigation of APOBEC-mediated R-loop mutagenesis in Jurkat cells. **A** Outline of the model system and the experiments performed. **B** Immunofluorescent detection of HSV-1 using an anti-HSV-1 glycoprotein B antibody (green). Cell membranes were labeled by the non-permeable vital stain Evans blue (magenta, pseudocolored). The bottom row shows zoomed-in views of infected cells from the top row. **C** Genome browser snapshot showing the DRIP and ChIP signals as well as the identified peaks. Seven representative regions of the HSV-1 genome are depicted, highlighting areas that accumulate C-to-T mutations, R-loops, and A3A and/or A3G binding sites (vertical lines). Data was normalized to input. **D** High-resolution genome browser view of C → T mutation hotspots in the HSV-1 genome. Five regions are highlighted and magnified to display local mutation patterns. (Color variations in IGV tracks (red, blue) reflect IGV’s default genotype display and do not represent quantitative variant allele frequencies.) **E** Proportions of all nucleotide substitution types (C → T, C → G, C → A, A → G, A → T, A → C) identified in the APOBEC ChIP samples and input samples. Data from two biological replicates were combined for the analysis. The ratio of C → T and C → G mutations was significantly higher than any other mutation type (two-proportion z-test, *p* < 0.00001). **F** Sequence logos derived from A3A and A3G ChIP-seq peak centers. Trinucleotide windows (yellow highlight) show a shared C-rich binding context with enrichment for a YC (Y = C/T) dinucleotide around the central cytosine. **G** Histogram depicting the distribution of distances between adjacent APOBEC mutations (in blue) and randomly distributed mutations (in yellow). The inset box plot shows that the median distance between APOBEC mutations is significantly less than what would be expected from a random distribution (Wilcoxon test *p*-value < 2.2 × 10^16^). **H** Anchor plot showing the enrichment of average R-loop (DRIP-seq) signal at the sites of identified APOBEC (C → T) mutations (*n* = 106) across the HSV-1 genome. This data was collected from ChIP-seq and DRIP-seq samples. The positions of mutations were extended by ± 10 nucleotides. Rep1 and Rep2 indicate independent biological replicates. **I** Left panel: Venn diagram showing the number of overlapping peaks for A3A, A3G, and R-loops in HSV-1-infected Jurkat cells. Right panel: Fold enrichment of C → T mutations within both the overlapping and distinct regions of the Venn diagram. The cells display the number of observed mutations relative to computer-simulated randomized (rnd) mutations (obs/rnd ratio). The warmth of the cells is proportional to the fold change, with 84.9% of C → T mutations occurring at the intersection of R-loops and A3A/A3G binding sites. Mutations are underrepresented in regions exclusive to R-loops and A3A/A3G binding sites

We selected A3A and A3G enzymes over other A3 family members to compare a predominantly nuclear (A3A) and a mostly cytosolic (A3G) APOBEC enzyme [9, 24] having distinct sequence preferences [25–27]. We adopted human Jurkat T lymphoblast cells as a primary model (Fig. 1A) because immune cells are natural targets for HSV-1 infection [28–34]. We first performed infective titration throughout the lytic phase of the HSV-1 cycle (Fig. 1B, Additional file 1: Fig. S1A) and measured cell viability to identify a post-infection time (12 h) that yielded sufficient levels of viral burden alongside minimal cell death. Subsequent molecular analyses were conducted during this phase of infection. Considering that herpesvirus infection can induce DNA damage [35–37], it was important to ensure the integrity of our DNA samples. Comet assay analysis of HSV-1-infected cells revealed no evidence of genome fragmentation compared to mock-infected cells (Additional file 1: Fig. S1B).

A3A and A3G protein expression increased in HSV-1-infected cells compared to mock controls (Additional file 1: Fig. S2A), which was accompanied by an increase in R-loop signal [38] at the single cell level (Additional file 1: Fig. S2B-D). Experimental techniques are in the figure legend. Combined RNase H/RNase A treatments confirmed that the S9.6 signal primarily represented RNA–DNA hybrids (Additional file 1: Fig. S3). These data provide the first evidence of A3A and A3G upregulation in response to HSV-1 infection, aligning with previous observations in other viruses [39, 40].

To explore the interplay between R-loops, A3 enzyme binding, and local mutation rates, we applied DNA–RNA immunoprecipitation sequencing (DRIP-seq) [41, 42] and ChIP-seq. DRIP-seq uncovered 34 R-loop peaks within the HSV-1 genome (Additional file 2: Table S3), while ChIP-seq analysis identified 52 A3A and 38 A3G binding sites, respectively (Fig. 1C, Additional file 2: Table S3). These profiles provide insights into the specific regions of the HSV-1 genome where R-loops and APOBEC enzymes interact. RNase H-treated control samples resulted in a significant reduction in the S9.6 signal in our DRIP experiments, confirming that the observed R-loops are specific. Some of the peaks identified by NGS were validated by DRIP-qPCR and ChIP-qPCR (Additional file 1: Fig. S4).

Regarding APOBEC3-mediated mutations in the HSV-1 genome, we detected 106 C → T changes that are the signature outcome of APOBEC cytidine deamination (Additional file 3), concentrated in a limited number of hotspot regions (Fig. 1D, Additional file 1: Table S1, Additional file 2: Table S3). These mutations were statistically enriched relative to other substitution types (two-proportion z-test, *p* < 0.00001; Fig. 1E). In addition, C → G and C → A changes are also attributed to cytidine deaminase activity in COSMIC signature SBS13 [43], further supporting an APOBEC origin of these hotspot mutations. Motif analysis of A3G and A3A ChIP peak summits revealed a shared C-rich binding context with enrichment for a YC dinucleotide (Y = C/T, [25–27]; Fig. 1F). This suggests that both enzymes are recruited to similar C-dense regions of the viral genome.

C → T mutations displayed a highly non-random, clustered distribution, with an average inter-mutational distance of ~ 20 nucleotides (Fig. 1G, Additional file 1. Table S1). This pattern is reminiscent of kataegis, i.e. localized “showers” of mutations typically attributed to clustered APOBEC activity [44]. Notably, C → T mutations were predominantly enriched in R-loop regions (Fig. 1H), emphasizing the mutagenic potential of R-loop structures in driving localized mutation patterns. However, R-loops accumulate APOBEC mutations only when they overlap with A3A and/or A3G binding sites (Fig. 1I). The majority of APOBEC-associated mutations (84.9%) occur in regions where R-loops and A3A/A3G binding sites coincide, whereas only a minority are detected in regions containing R-loops alone or A3A/A3G binding alone. These findings support a model in which R-loops create accessible substrates in the viral genome for A3-mediated deamination. However, this pattern may also be shaped by post-binding events: lesions arising at A3A/A3G-bound sites outside R-loop regions may be efficiently repaired by host and/or viral DNA repair pathways [45, 46], and APOBEC enzymes may additionally be relocalized away from viral DNA by herpesviral evasion mechanisms [3, 5]. Thus, the preferential detection of APOBEC mutations near R-loops may reflect a combination of enhanced substrate accessibility, altered local repair kinetics, and differences in APOBEC residence time at viral DNA.

The identified C → T mutations were concentrated in coding sequences, displaying a 1.5-fold enrichment on coding strands (43 mutations) compared to template strands (28 mutations), with more than 5.95-fold increase in mutation density per unit length relative to other genomic regions (see Methods). Affected genes—including *UL36*, *UL15*, *UL46*, and *US4*—encode proteins critical for viral replication and immune evasion. Notably, *UL36*, which encodes an essential tegument protein with ubiquitin-specific protease activity [47–49], harbored multiple premature stop codons (Additional file 1: Fig. S5A and Fig. S10), that truncate its C-terminal domain, a region essential for viral DNA replication and transport [6, 50]. Three-dimensional structural modeling confirms these APOBEC-induced alterations and their potential impact on protein integrity (Additional file 1: Fig. S5B).

Similar missense mutations were detected in *UL15* (terminase large subunit essential for DNA cleavage and packaging *UL46* (tegument protein targeted by HSV-specific CD4 + and CD8 + T cells [51], and *US4* (glycoprotein G), which modulates virulence [52] (Additional file 1: Fig. S5C-E and Fig. S10). While not all mutant variants necessarily dominate the viral population, these findings demonstrate that APOBEC3 activity can introduce functionally damaging mutations into genes essential for HSV-1 replication and immune defense.

**Fig. 2:**
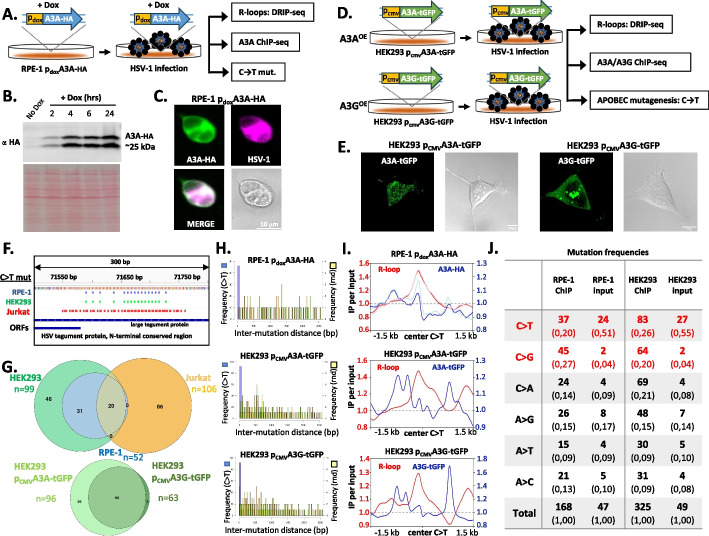
Orthogonal APOBEC3 overexpression systems link HSV-1 mutagenesis to R-loops across multiple cell types. Schematic of the two complementary gain-of-function approaches used to validate the APOBEC–R-loop connection: a doxycycline-inducible A3A-HA construct in RPE-1 cells and CMV-driven A3A-tGFP or A3G-tGFP in HEK293-AD cells. **A** Experimental design: following doxycycline (dox) induction, RPE-1 A3A-HA cells were infected with HSV-1 and subjected to DRIP-seq (R-loops), anti-HA ChIP-seq (A3A binding), and viral mutation analysis. **B** Western blot with anti-HA antibody showing time-dependent accumulation of A3A-HA after doxycycline addition; the 4-h induction point was used for all NGS-based assays. Total protein staining is shown as a loading control. **C** Representative immunofluorescence image of doxycycline-treated, HSV-1–infected RPE-1 cells, illustrating robust A3A-HA expression (green), viral antigen staining (magenta), and their overlap in merged channel. **D** Constitutive A3A-tGFP and A3G-tGFP expression in HSV-1–infected HEK293-AD cells. Schematic of CMV-driven A3A-tGFP and A3G-tGFP constructs used to generate stable HEK293-AD lines; these cells were infected with HSV-1 and processed for DRIP-seq, ChIP-seq with anti-tGFP, and mutation profiling. **E** Live-cell confocal images showing A3A-tGFP and A3G-tGFP distributions in HEK293-AD cells. **F** Representative genome-browser view of a 300-bp HSV-1 region illustrating APOBEC-type mutations identified in RPE-1 (blue), HEK293-AD (green), and Jurkat (red) cells. Mutations cluster in discrete hotspots that recur across independent experimental platforms. **G** APOBEC mutation overlap across cell types. Upper panel: Venn diagram of C → T changes in Jurkat, RPE-1 A3A-HA, and HEK293-AD A3A/A3G-tGFP cells, showing a statistically significant shared subset of hotspots, with the strongest overlap between the two epithelial cell systems. Lower panel: Venn diagram comparing C → T mutations in HEK293-AD A3A-tGFP and A3G-tGFP cells. Nearly all C → T mutations in A3G-tGFP cells coincide with those in A3A-tGFP cells, whereas A3A-tGFP generates additional sites not targeted by A3G-tGFP. **H** Inter-mutation distance analysis. Histograms depict the distribution of distances between adjacent APOBEC-type mutations (blue) compared with an equal number of randomly positioned mutations (yellow) for RPE-1 A3A-HA (top), HEK293-AD A3A-tGFP (middle), and HEK293-AD A3G-tGFP (bottom) cells. In all cases, observed inter-mutation distances are markedly shorter than random (Wilcoxon test *p* < 2.2 × 10⁻^1^⁶), indicating strong clustering of APOBEC edits. **I** Colocalization of APOBEC binding with R-loops at C → T mutations. Anchor plots show normalized DRIP-seq signal (R-loops, red) and APOBEC3 ChIP-seq signal (A3A-HA and A3A/A3G-tGFP, blue) centered on C → T mutation sites extended by ± 1.5 kb. In RPE-1 cells, the A3A-HA and R-loop peaks coincide, whereas in HEK293-AD cells the A3A/A3G-tGFP signals form twin peaks flanking the central R-loop summits, consistent with APOBEC binding to ssDNA bordering the RNA–DNA hybrid within the broader R-loop domain. **J** Substitution mutation spectra in APOBEC ChIP and input samples. Table summarizing counts and frequencies of base-substitution types in HSV-1 genomes recovered from A3A/A3G ChIP and matched input DNA. C → T transitions and C → G transversions are enriched compared with other substitution classes (two-proportion z-test *p* < 0.00001)

To validate the APOBEC–R-loop–linked mutagenesis observed in HSV-1–infected Jurkat cells, we established two independent gain-of-function systems that differ in cell type, expression mode, epitope tag, and detection antibody. In human retinal pigment epithelium (RPE-1) cells, we generated a doxycycline-inducible A3A-HA construct (Fig. 2A). Western blot analysis showed rapid A3A-HA induction after doxycycline treatment, with robust, homogeneous levels by 4 h; this time point was therefore selected for further analysis (Fig. 2B). Microscopy confirmed strong nuclear localization of A3A-HA and its presence in HSV-1–infected cells (Fig. 2C, Additional file 1: Fig. S6A), indicating that A3A-HA efficiently accesses nuclear viral genomes.

In parallel, we established constitutive overexpression systems in HEK293-AD cells using CMV-driven turboGFP-tagged A3A and A3G (Fig. 2D). Live-cell imaging revealed that A3A-tGFP was localized predominantly to the nucleus, whereas A3G-tGFP was largely cytoplasmic (Fig. 2E, Additional file 1: Fig. S6B, C). HSV-1 infection proceeded efficiently in both lines, demonstrating that the constructs do not block viral entry or perturb cell physiology.

Genome-wide mutational analysis across Jurkat, RPE-1 A3A-HA, HEK293-AD A3A-tGFP, and HEK293-AD A3G-tGFP systems revealed that C → T mutations cluster in discrete hotspot regions of the HSV-1 genome (Fig. 2F, Additional file 1: Table S1). Comparison of mutation positions showed substantial overlap between cell lines, with the highest concordance between the two epithelial systems (RPE-1 and HEK293-AD; Fig. 2G, upper panel). In HEK293-AD cells, C → T mutations detected in A3G-tGFP–expressing cells were almost entirely (~ 99%) shared with those identified in A3A-tGFP–expressing cells, whereas A3A-tGFP cells harbored additional unique hotspots (Fig. 2G, lower panel). These patterns indicate that both enzymes converge on the same preferred regions of the viral genome, with A3A generating more dispersed edits, consistent with its higher catalytic activity and more stochastic mutagenesis.

Inter-mutation distance analysis further supported non-random, processive APOBEC activity. In all three overexpression systems, the observed distances between adjacent APOBEC-type mutations were markedly shorter than expected for an equal number of randomly distributed mutations along the HSV-1 genome (Wilcoxon test, *p* < 2.2 × 10⁻^16^; Fig. 2H), demonstrating strong clustering of these edits.

Integration of DRIP-seq and ChIP-seq data revealed a tight spatial relationship between R-loop formation, APOBEC3 binding, and mutation sites (Fig. 2I). In RPE-1 cells expressing inducible A3A-HA, the R-loop signal and A3A-HA ChIP profile peaked at the positions of C → T mutations, indicating that A3A-HA accumulates within R-loop cores. By contrast, in HEK293-AD cells expressing A3A-tGFP or A3G-tGFP, APOBEC ChIP signals flanked the central R-loop peak bilaterally. This difference likely reflects distinct expression regimes (acute induction versus constitutive overexpression), or an artefact of alternative tags (HA versus tGFP, which may influence binding characteristics. It is possible that the inducible RPE-1 system may capture A3A at early-stage recruitment to nascent R-loops, whereas constitutively expressing HEK293-AD cells allow APOBEC3 to accumulate at R-loop boundaries where flanking single-stranded DNA is exposed. Regardless of these differences, all systems consistently positioned APOBEC3 occupancy and mutagenesis within or adjacent to R-loop regions.

Quantitative analysis confirmed enrichment of the canonical APOBEC mutational signature in ChIP-enriched HSV-1 DNA (Fig. 2J). In addition to C → T mutations, C → G transversions—consistent with error-prone repair of APOBEC-induced deoxycytidine lesions—were significantly overrepresented relative to other substitution types and to input controls (*p* < 1 × 10⁻^5^). This composite signature links the observed HSV-1 hotspot mutations at R-loop–associated regions to APOBEC3 activity.

## Conclusions

We show that R-loops and APOBEC3 binding together promote targeted mutagenesis in HSV-1, with R-loops providing accessible substrates for cytidine deamination. Mutagenesis requires both elements: R-loops alone are non-mutagenic, while A3 binding in the absence of an R-loop does not generate detectable mutations, possibly because such lesions are efficiently repaired and/or A3 enzymes are relocalized away from viral DNA. This dual requirement explains why many HSV-1 genes remain unaffected under our conditions. The acute, hotspot-based nature of APOBEC3 editing indicates that R-loop formation creates discrete vulnerabilities in viral genomes that host enzymes can exploit. These focal R-loop–linked sites may represent candidate targets for suppressing HSV-1 and potentially other viral pathogens [53]. Limitations of the study are discussed in Additional file 3: Supplementary text.

## Methods

### Cell lines and HSV-1 infection

Jurkat human T lymphoblast cells were obtained from ATCC-J.RT3-T3. Human embryonic kidney cells (HEK293-AD) were obtained from Agilent (240085, AD-293). Human retinal pigment epithelium (RPE-1) cells were a gift from Dr. Priyanka Verma (ID: CVCL_4388, Cellosaurus). Cell line authentication was not performed in our laboratory. All cell lines were regularly tested and confirmed to be free of mycoplasma contamination throughout the study. Jurkat cells were cultured in RPMI-1640 medium (Merck KGaA, Darmstadt, Germany) containing fetal bovine serum (10%), streptomycin (100 μg/ml), penicillin (100 units/ml), and L-glutamine (2 mM). RPE-1 p_dox_A3A-HA cells were generated by lentiviral transduction with the doxycycline-inducible pFLRU-A3A lentivector with a Thy1.2 selection marker. Thy1.2-positive cells were isolated by magnetic column using Thy1.2-biotin beads (CD90.2, Miltenyi). Thy1.2 positivity was determined by flow cytometry, and a stable positive population was maintained (> 95% Thy1.2 positive). Single-cell clones were obtained by serial dilution, with a ratio of 1 cell for every 3 wells of a 96-well plate. Single-cell clones were grown in DMEM supplemented with 20% tetracycline-free FBS and 1% penicillin/streptomycin until clones expanded, then were maintained in DMEM supplemented with 10% tetracycline-free FBS and 1% penicillin/streptomycin. The selected RPE-A3A clone was tested for A3A expression with doxycycline induction (0.25 µg/mL) and Western blot analysis for the C-terminal HA tag on A3A. HEK293-AD p_CMV_A3A-tGFP and p_CMV_A3G-tGFP cells were established by stable transfection with APOBEC3A-tGFP and APOBEC3G-tGFP overexpression plasmids (OriGene, RG220995 and RG206821, respectively; pCMV6-AC-GFP backbone with C-terminal turboGFP tag and neomycin resistance) using PEI (25 kDa, branched; 100 µL of a 4.5 mg/10 mL stock) according to standard protocol. 48 h post-transfection, cells were placed under selection with G418 (neomycin; 800 µg/mL) for 14 days, with medium changed every 2–3 days until discrete resistant colonies appeared, which were screened for A3A-tGFP or A3G-tGFP expression by fluorescence microscopy. Vero African green monkey kidney cells (ATCC-CCL-81) were employed for virus propagation, maintained in Minimum Essential Medium. The KOS1 strain of HSV-1 (ATCC-VR-1493; LOT: 59681562) was cultured in Vero cells, which were also mock-infected, and the harvested suspension was further used for mock infection of Jurkat, RPE-1 p_dox_A3A-HA and HEK293-AD p_CMV_A3A-tGFP and HEK293-AD p_CMV_A3G-tGFP cells. The infective titer of HSV-1 was calculated as 50% tissue culture infectious dose (TDID50) by the Spearman-Kärber algorithm [54, 55]. The KOS1 strain of HSV-1 was employed to infect the cell lines at a multiplicity of infection (MOI) of 5. HSV-1 infection of RPE-1 p_dox_A3A-HA cells was performed 2 h after the addition of doxycycline (dox) to induce A3A-HA expression, and dox was maintained throughout the experiment (12 h post-infection). Following incubation of the cells with the virus for 2 h, excess HSV-1 was removed, and the cells were cultivated for downstream analysis. We fixed cells in 1% formaldehyde (Thermo Fisher Scientific, Pierce™ 16% Formaldehyde (w/v), Methanol-free,) at various time points post-infection.

### ChIP and DRIP

Twenty million cells, either infected with HSV-1 or treated with Vero cell extract (mock control), were grown to near-confluence in T175 flasks and crosslinked with 1% methanol-free formaldehyde for 10 min at room temperature. Crosslinking was quenched with glycine (final concentration 500 mM, pH 6) for 5 min. Cells were washed three times with ice-cold PBS, pelleted by centrifugation (1000 × g, 5 min, 4 °C), collected in ChIP lysis buffer by scraping, and stored at –80 °C until processing. Sonication, immunoselection and immunoprecipitation were performed following the protocol in [56]. Briefly, Chromatin was sheared by sonication (4 × 5 cycles, 30 s ON/OFF, low intensity). Cell debris was pelleted, and the supernatant containing fragmented chromatin was transferred to fresh tubes. Fragment size (target range 200–500 bp) was verified by agarose gel electrophoresis after overnight crosslink reversal at 65 °C with RNase A and proteinase K treatment, followed by column-based DNA purification (Zymo). Two percent of the sample was kept as input DNA. For ChIP, 8 µg of rabbit polyclonal anti-APOBEC3A (D-23) (Sc-130688) and anti-APOBEC3G (H-63) (Sc-48820) rabbit polyclonal antibodies were used (Jurkat cells), while the anti-HA rabbit polyclonal antibody (Abcam ab9110) was used for RPE-1 p_dox_A3A-HA cells and anti-tGFP mouse monoclonal turboGFP antibody (Origene, clone OTI2H8) for HEK293-AD p_CMV_A3A-tGFP and p_CMV_A3G-tGFP cells. RNA–DNA hybrid precipitation (DRIP) was conducted on the same samples used for ChIP, utilizing the S9.6 monoclonal antibody, following the procedure outlined in Protocol 5 in [41]. Genomic DNA was isolated using the Nucleospin Tissue Kit (Macherey–Nagel) without RNase A treatment and eluted in low-EDTA TE buffer. Six micrograms of gDNA per sample was sonicated (Diagenode Bioruptor; 3 × 5 min, low power, 30 s ON/OFF). Fragmentation efficiency was assessed by agarose gel electrophoresis after RNase A treatment. For RNase H controls, 6 µg sonicated DNA was digested with RNase H (1.33 µl per µg DNA; 5000 U/ml, NEB) overnight at 37 °C, followed by DNA purification. Protein G magnetic beads were blocked with 0.5% BSA/PBS/EDTA and incubated with 15 µg S9.6 antibody per 50 µl beads for 4 h at 4 °C with rotation. Sonicated DNA (100 µl) was diluted to 1 ml in DRIP buffer with protease inhibitors and incubated with antibody-coated beads overnight at 4 °C. Beads were washed three times each with DRIP buffer and TE buffer at 4 °C, then eluted in IP elution buffer containing proteinase K for 2 h at 55 °C. DNA was purified using Ampure XP beads (2 × ratio), eluted in low-EDTA TE, and quantified using a Qubit dsDNA HS assay.

### NGS and bioinformatics

Preparation of NGS libraries and sequencing were performed as described [56–58] using Illumina’s TruSeq ChIP Sample Preparation method. Libraries were run on an Illumina HiSeq 2500 instrument applying 2 × 125 paired-end sequencing, and Illumina NovaSeq X Plus PE150 (Novogene Ltd.). FastQC v0.11.9 was used to assess the quality of raw reads. DRIP-seq/ChIP-seq reads from the Jurkat cell line were aligned to the HSV-1 KOS reference sequence [59] using bwa-mem. Default settings were used for DRIP-seq samples, while for the A3A/A3G ChIP-seq samples, a mismatch penalty of -B 1 was applied. For the RPE-1 and HEK293 cell lines, we employed two complementary alignment strategies. First, DRIP-seq and ChIP-seq reads were aligned to the HSV-1 KOS1 reference genome using Bowtie2 [60] with default parameters to determine the genomic distribution of R-loops, A3A, and A3G. Subsequently, we performed an additional alignment using bwa-mem with a more permissive mismatch setting (-B 0) to enable the detection of APOBEC-associated mutations that would otherwise be penalized or lost during standard alignment. Samtools v1.10 [61] was used to filter properly-paired reads with a mapping quality score higher than 30 and to remove PCR duplicates. RPKM (Reads Per Kilobase per Million mapped reads) normalized coverage files were generated (.bigwig and.bedgraph) with 20 bp resolution using Deeptools bamCoverage v3.5.140 [62]. Metaplots were created for 10 bp bins using computeMatrix and were plotted using deepTools. MACS2 [63] was used with a cutoff of 1.3 (*p* = 0.05) to identify DRIP and ChIP peaks in input-normalized samples. APOBEC mutations were identified using the Freebayes tool [64]. For this, we generated VCF files from BAM files with default parameters and identified initial variants that were subsequently filtered by quality and coverage criteria: only variants with a QUAL score of at least 10 and a DP value of at least 10 were retained. To focus on APOBEC mutations, we filtered for G → A or C → T mutations. The mutation database from DRIP, ChIP, and input samples were unified in order to remove redundancies. This filtering process resulted in 106/99/52 C → T mutations in Jurkat, HEK293-AD, and PRE-1 cells, respectively, which are characteristic of APOBEC activity. NGS data were visualized in Integrative Genomics Viewer (IGV). To identify the consensus binding motifs associated with A3A and A3G ChIP-seq peaks, we used the MEME [65] motif discovery tool (v5.4.1, offline mode). MEME determined the frequency distribution of nucleotides at each position, producing a position weight matrix (PWM) and the most probable motif. The likelihood of the identified motifs was statistically evaluated using the Likelihood Ratio Test (LRT). P-values were calculated and subsequently converted to E-values to account for sequence number corrections. qPCR was used to validate DRIP-seq and ChIP-seq peaks on selected HSV-1 regions, with primers listed in Additional file 1: Table S2. Reactions were performed using the LightCycler 480 SYBR Green I Master reagent (Roche, cat#04887352001) under the following cycling conditions: an initial denaturation at 95 °C for 1 min, followed by 40 cycles of 95 °C for 15 s, 55 °C for 1 min, and a final extension at 55 °C for 1 min. The experiments were conducted using a QuantStudio 12 K Flex system.

### Immunofluorescence and microscopy

HSV-1 antigen in infected cells was detected by indirect immunofluorescence. Following methanol fixation, cells were blocked with 1% BSA in PBS, then incubated with primary antibodies in blocking buffer for 1 h at room temperature: mouse monoclonal anti-HSV-1/HSV-2 gB [10B7] (ab6506, Abcam) at 1:100. After four 5-min PBS washes, cells were incubated with Alexa Fluor 488-conjugated goat anti-mouse (A11029, Invitrogen) secondary antibodies in blocking buffer for 1 h at room temperature in the dark. Samples were washed four times with PBS before imaging. For A3A/A3G and R-loop dual-labelling, Jurkat cells were first suspended in low melting point agarose (1%) at 37 °C, prepared in PBS, spread into multichambered microscopic slides (~ 30,000 cells/well), and washed with PBS. Cells were lysed for 10 min on ice in a buffer containing 2 M NaCl and 1% (v/v) Triton X-100, prepared in PBS/5 mM EDTA. After lysis, the samples were incubated with BSA (5 mg/mL) dissolved in PBS/5 mM EDTA for 30 min, on ice. Detection of R-loops was achieved by incubating the samples with the S9.6 monoclonal mouse antibody and a secondary anti-mouse Alexa488 antibody. The RNA–DNA hybrid-specific S9.6 monoclonal antibody was produced from the Hb-8730 mouse hybridoma cell line (purchased from ATCC) using standard protein A/G affinity purification [66]. The purified antibody was stored in PBS containing 0.05% (w/v) sodium azide. A3A and A3G enzymes were labeled using the anti-APOBEC3A (D-23) (Sc-130688, Santa Cruz Biotechnology, Inc) rabbit polyclonal antibody and the anti-APOBEC3G (H-63) (Sc-48820, Santa Cruz Biotechnology, Inc) rabbit polyclonal antibody, respectively, followed by a goat anti-rabbit Alexa647 antibody (Thermo Fisher Scientific). For RNase H and RNase A digestion, samples were incubated under identical conditions (37 °C for 2 h) with either 8 μl of *E. coli* RNase H (5000 U/ml; NEB) in a total volume of 100 μl RNase H-reaction buffer, or 3 μl of RNase A (Thermo Fisher Scientific, EN0531) in TE buffer. Imaging was performed on a Zeiss Axiovert 200 M confocal laser scanning microscope. The images were analyzed using ImageJ/Fiji software (https://imagej.net/software/fiji/). For each sample, 1 µm optical stacks were acquired. For visualization, a z-projection was applied to the optical slices in each channel, creating single stacks that were presented in the figures.

### Automated microscopy (LSC)

LSC imaging was performed by an iCys Research Imaging Cytometer (CompuCyte, USA). RNA–DNA hybrids and APOBEC3A/3G enzymes were labeled according to the protocol detailed in the previous section. The Alexa 488 dye was excited by the 488 nm line of an Argon ion laser, while the Alexa 647 dye was excited by the 633 nm line of a HeNe laser. Fluorescent signals were collected using a UPlan FI 20 × objective with a numerical aperture (NA) of 0.5, using 510/521 nm and 530/530 nm filters for Alexa 488, and a 650/LP nm filter for Alexa 647. Integral fluorescence intensities were measured using the iCys 7.0 software, expressing the sum of fluorescence intensities of pixels corresponding to each cell.

### Comet assay

Cells were embedded in low melting-point agarose (1%) made in 10 mM Tris–HCl/1 mM EDTA, pH 8.0, and spread in microscopic chambers. Where indicated, cells were exposed to the topoisomerase II inhibitor etoposide (40 μM) for 1 h to induce DNA fragmentation. Samples were lysed in 50 mM EDTA/0.5% Triton X-100/0.2% Sarkosyl/2 M NaCl/10 mM Tris–Cl, pH 8/10% DMSO buffer for 5 min, on ice. Agarose gel electrophoresis was performed in alkaline conditions (1 mM EDTA/300 mM NaOH) for 1 h at 4 °C. The alkaline pH was neutralized for 5 min in a buffer containing 10 mM EDTA and 1 M Tris–HCl, pH 8, and then stained with propidium iodide. Comets were visualized using automated microscopy (LSC) and quantified with OpenComet software [67].

### Western blot

Whole-cell extracts were prepared from 1 ml of formaldehyde-fixed Jurkat cells (10⁷ cells/ml) under mock-treated or HSV-1-infected conditions. Cells were lysed in 100 μl of 2 × Laemmli buffer at 95 °C for five minutes, followed by neutralization with 5–10 μl of unbuffered 1 M Tris. Protein separation was performed by 14% SDS-PAGE, and proteins were subsequently transferred to nitrocellulose membranes (Merck, A-21236 Amersham™ Protran® Western blotting membranes) using standard electroblotting procedures. The APOBEC3A (A3A) and APOBEC3G (A3G) enzymes were detected using rabbit polyclonal antibodies: anti-APOBEC3A (D-23) (Sc-130688, Santa Cruz Biotechnology, Inc.) and anti-APOBEC3G (H-63) (Sc-48820, Santa Cruz Biotechnology, Inc.). Detection was performed with an Goat anti-Rabbit IgG (H + L) Secondary Antibody, HRP (1:100,000; Amersham) and the ECL Prime Detection System (GE Healthcare). Unedited western blots are shown in Additional file 1: Figs. S8-S9.

### Protein sequence alignment and structural modeling

Amino acid sequence alignments of wild-type and mutant HSV-1 proteins were carried out using the MUSCLE tool [68]. The complete aligned sequences are available in Additional file 1: Fig. S10. To generate a 3D model of the UL36 protein, we employed AlphaFold2 [69] through ColabFold [70]. In the resulting 3D structure, the position of the C-terminal truncation caused by APOBEC mutations was highlighted.

### Mutational burden analysis

We quantified the number of C → T mutations per 1,000 bp in *UL36*, *UL15*, *UL46*, and *US4* genes. Then, we established a null model for comparison by randomly sampling 1,000 positions from the HSV-1 genome, excluding the regions of these four genes. The null distribution was generated by repeating the random sampling 50 times and averaging the results. We obtained a background mutation density of 0.66 mutations per 1,000 bp, while the observed specific mutation density for *UL36*, *UL15*, *UL46*, and *US4* was 3.93 mutations per 1,000 bp, which is 5.95 times higher than the background rate.

## Supplementary Information

Additional file 1: Supplementary Figures S1-S9 and Supplementary Tables S1-S2.

Additional file 2: Supplementary Table S3 containing the identified C → T mutations and genomic positions of ChIP-seq and DRIP-seq peaks.

Additional file 3: Supplementary text about the limitations of the study.

## Data Availability

NGS datasets generated in this study have been deposited in the Gene Expression Omnibus (GEO) under accession numbers GSE277995 [71], GSE277996 [72], GSE284474 [73], and GSE312648 [74]. Microscopy image data have been deposited in Zenodo [75]. Shell scripts used for data processing have also been deposited in Zenodo [76].
