# Supplementary material for: Herpes simplex virus type 1 R-loops are targets for APOBEC-mediated mutagenesis
Source: Genome Biol. 2026 Apr 14;27:169. doi: 10.1186/s13059-026-04078-y (PMC13185267; doi:10.1186/s13059-026-04078-y)
Supplement: Supplementary file 1 — Additional file 1: Supplementary Figures S1-S9 and Supplementary Tables S1-S2. [file 13059_2026_4078_MOESM1_ESM.pdf]

Additional file 1

Herpes simplex virus type 1 R-loops are targets for APOBEC-mediated mutagenesis

Márton Miskei, Dóra Varga, Lilla Hornyák, Éva Sipos, Éva Nagy, Qiuzhen Li, Zsolt Karányi, Zoltán Szabó, Rachel DeWeerd, Abby M Green, Dávid Szüts, Eszter Csoma, Lóránt Székvölgyi

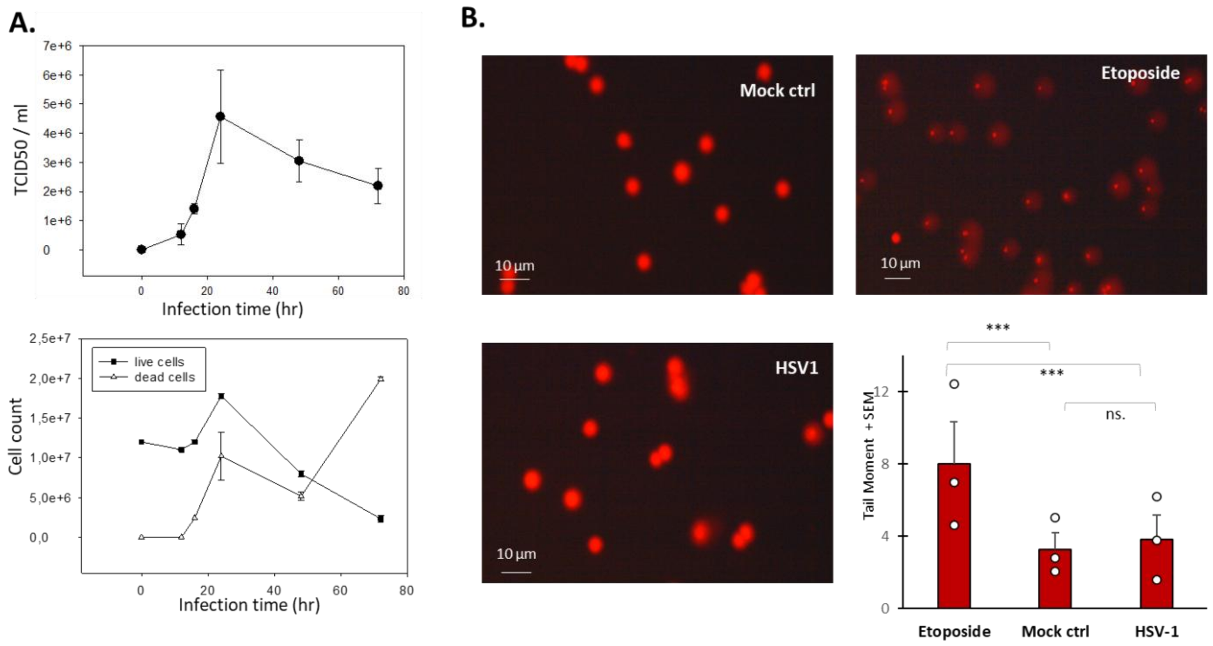

Fig. S1. A. Infective titration and cell viability of HSV-1 infected Jurkat cells. Top panel: each point on the resulting growth curve represents the average titer calculated from three independent experiments for each treatment or time point. Bottom panel: the number of live and dead cells as a function of infection time. Cell viability was determined by trypan blue exclusion method: cells taking up trypan blue were considered non-viable. B. Detection of DNA damage by alkaline comet assay in HSV-1 infected and Vero mock-treated cells. As a positive control, cells were treated with the topoisomerase II inhibitor etoposide (40 µM for 1 hour). Representative images are shown. The DNA was stained with propidium iodide. Tail moments were quantified by the OpenComet software. *** means a statistically significant difference (p<0,0001). ns.: non-significant. TCID50: 50% tissue culture infectious dose.

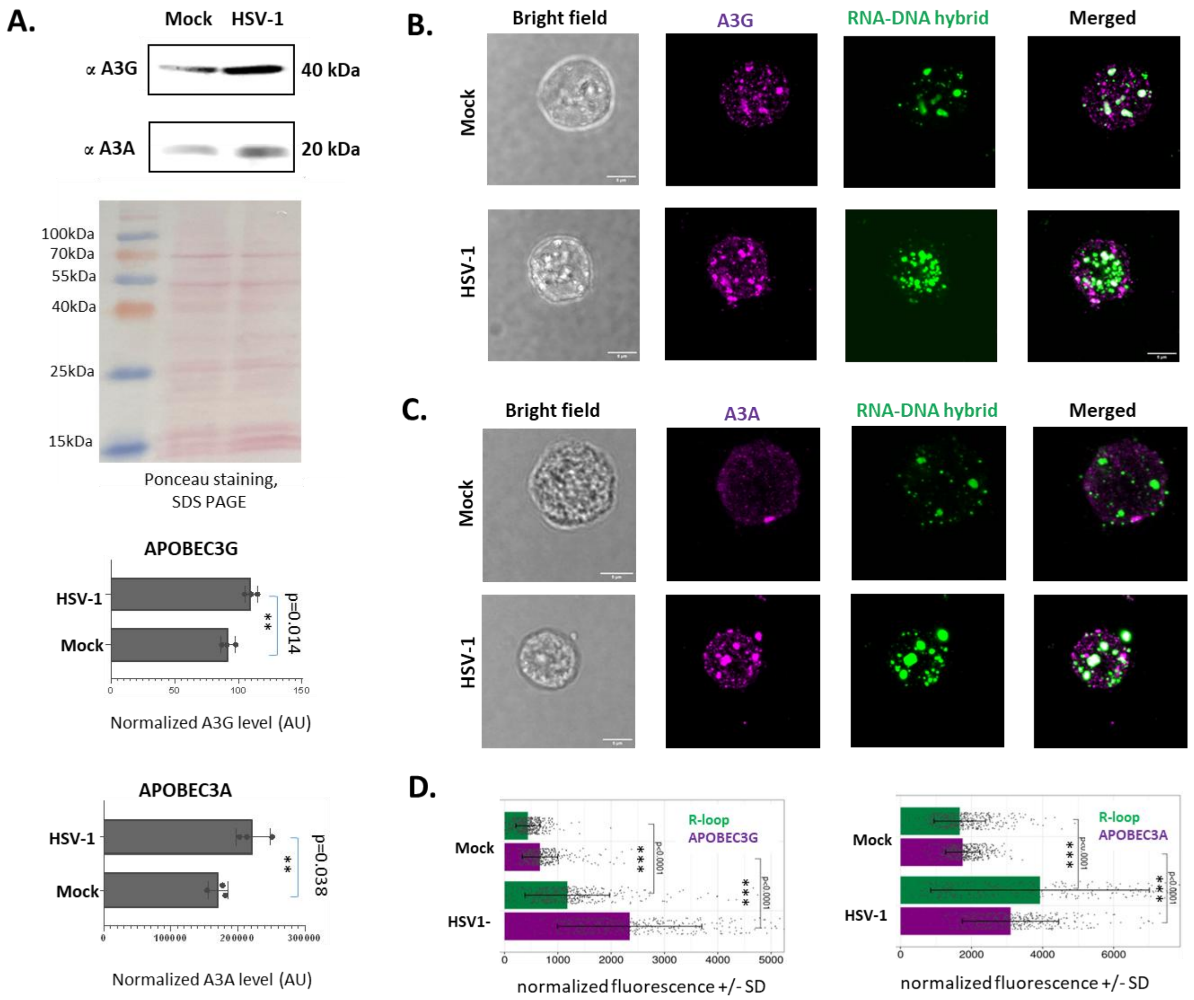

Fig. S2. APOBEC3A and APOBEC3G enzymes are upregulated during HSV-1 infection. (A) Western blot analysis of APOBEC3G and APOBEC3A expression. The antibodies detected a dominant band at the expected molecular weights of A3A (20 kDa) and A3G (40 kDa). Upregulation of A3G and A3A enzymes upon HSV-1 infection is significant (Mann-Whitney U test). Western blot band intensities were quantified and normalized to total protein levels, as determined by Ponceau red staining. The quantification results are displayed below the blot. (B-D) Immunofluorescence labeling of R-loop structures and A3A/A3G enzymes in HSV-1 infected Jurkat cells. (A) Representative confocal laser scanning microscopic image of HSV-1-infected and mock-treated Jurkat cells (with Vero cell extract). The cells were co-labeled using anti-APOBEC3G and anti-RNA-DNA hybrid (S9.6) antibodies to visualize the presence and overlap of A3G enzymes and R-loop structures. A total of 50 cells were analyzed. (C) This panel replicates the experiment described in panel A, substituting A3G with A3A. (D) Quantification of the fluorescent signals from the same samples using automated microscopy (LSC). Results demonstrate that both R-loop and A3A and A3G levels are significantly increased in HSV-1 infected cells compared to mock controls. A minimum of 5,000 cells were analyzed, and statistical significance is indicated (Student's t-test, significance level: 0.05).

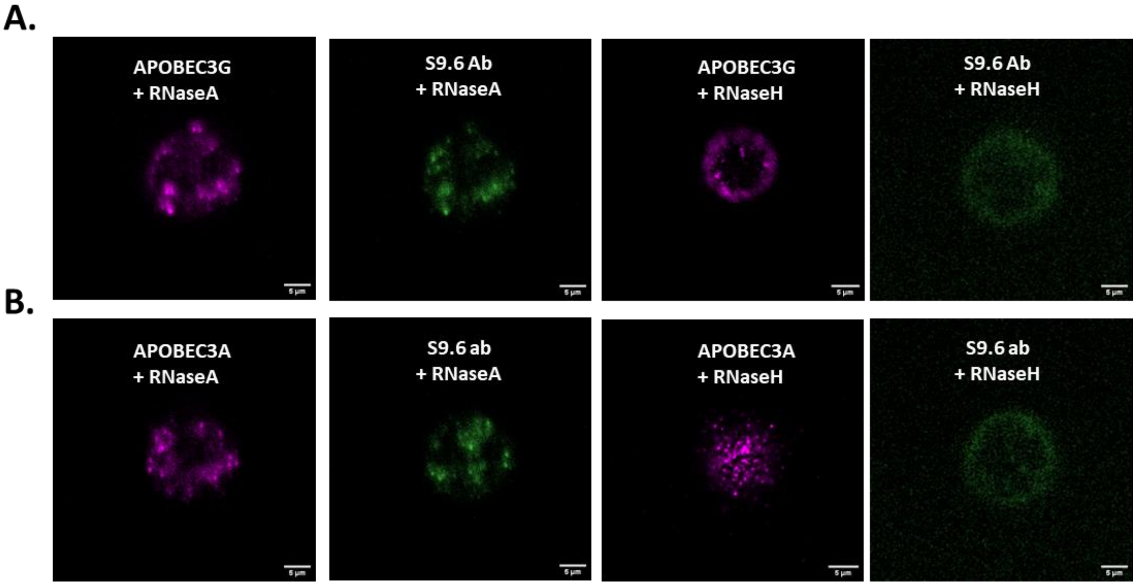

Fig. S3. Microscopic analysis of HSV-1-infected Jurkat cells treated with distinct ribonucleases. RNase A and RNase H were used to selectively degrade single-stranded RNA and RNA-DNA hybrids, respectively. RNA-DNA hybrids are visualized with the S9.6 antibody (green), while the (A) APOBEC3G and (B) APOBEC3A enzymes are labeled with specific antibodies (magenta). Representative images are displayed. Scale bar: 5 µm.

A.

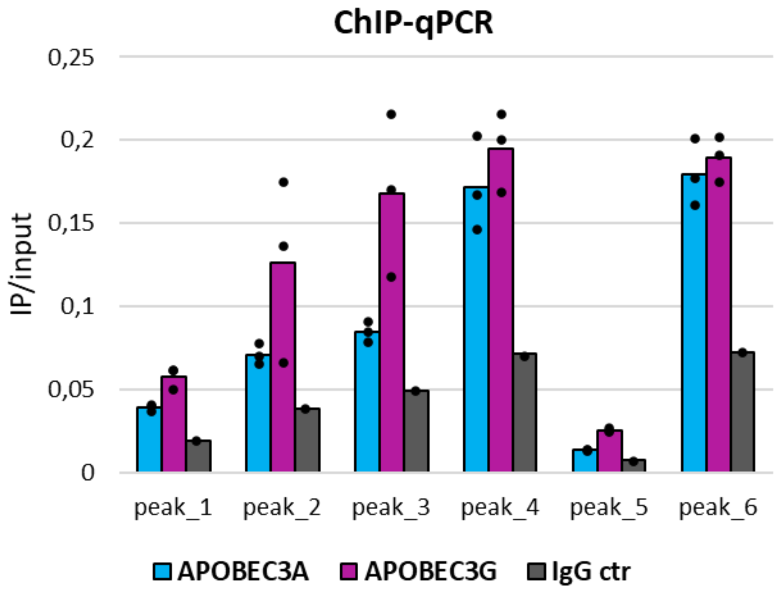

B.

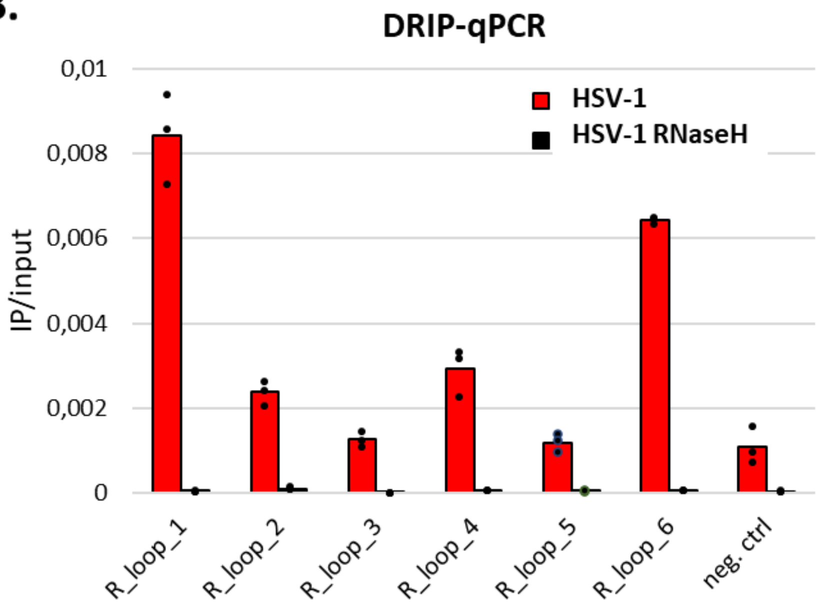

Fig. S4. qPCR validation of ChIP-seq and DRIP-seq data. Six peaks were selected for the analysis. For ChIP samples, IgG was included as a no-antibody control to account for background signal, with all peaks showing significant enrichment compared to IgG. For DRIP validation, RNase H-treated samples served as a negative control, exhibiting baseline S9.6 signal and confirming the specificity of S9.6 immunoprecipitation. Additionally, a control site from the genome (*ZNF554*) [1] was included.

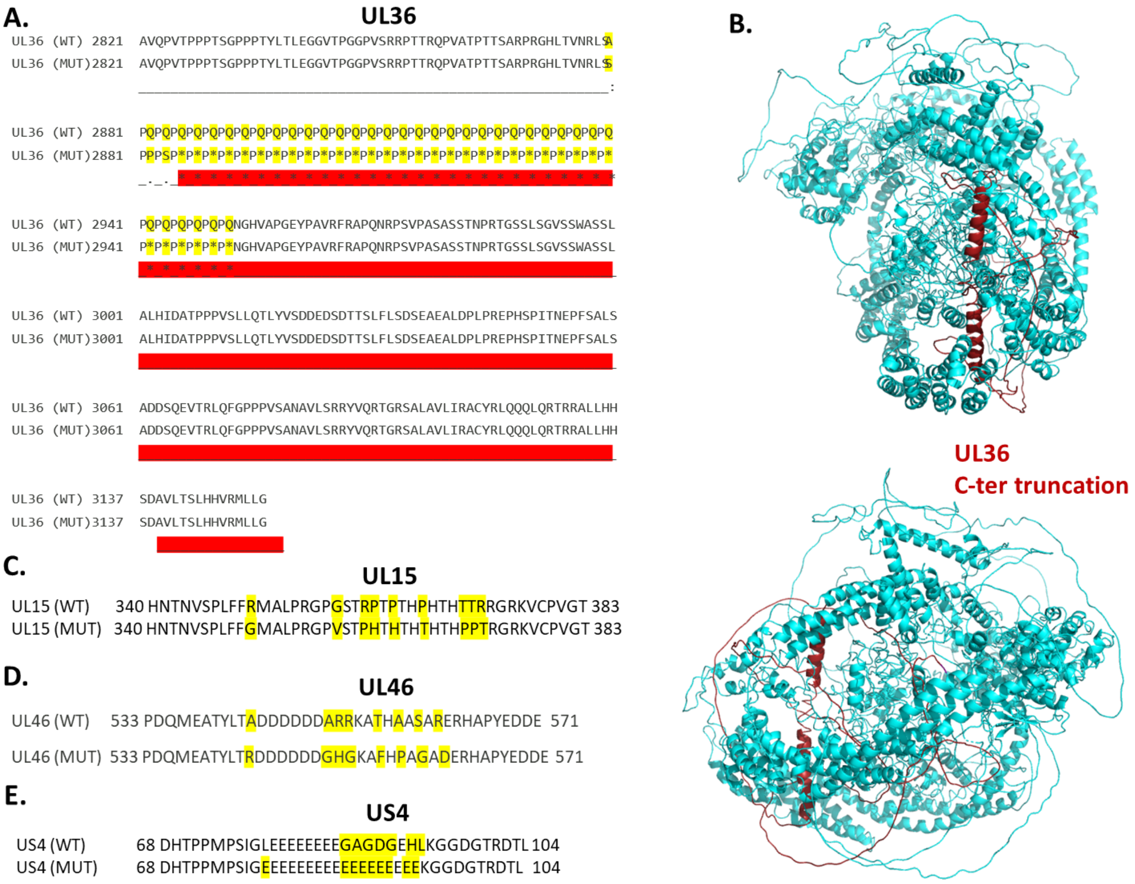

Fig. S5. APOBEC-induced mutagenesis and resulting amino acid changes in key HSV-1 proteins. A. Amino acid sequence alignment of wild-type and mutant UL36 protein highlighting C-terminal truncation. Stars (*) indicate premature stop codons. Amino acid changes are highlighted in yellow. B. 3D structural model of UL36 protein showing the position of C-terminal truncation caused by APOBEC mutations. The truncated (missing) part of the protein is highlighted in dark red. C-E. Amino acid sequence alignment of wild-type and mutant HSV-1 proteins (UL15, UL46, US4) highlighting missense mutations (yellow).

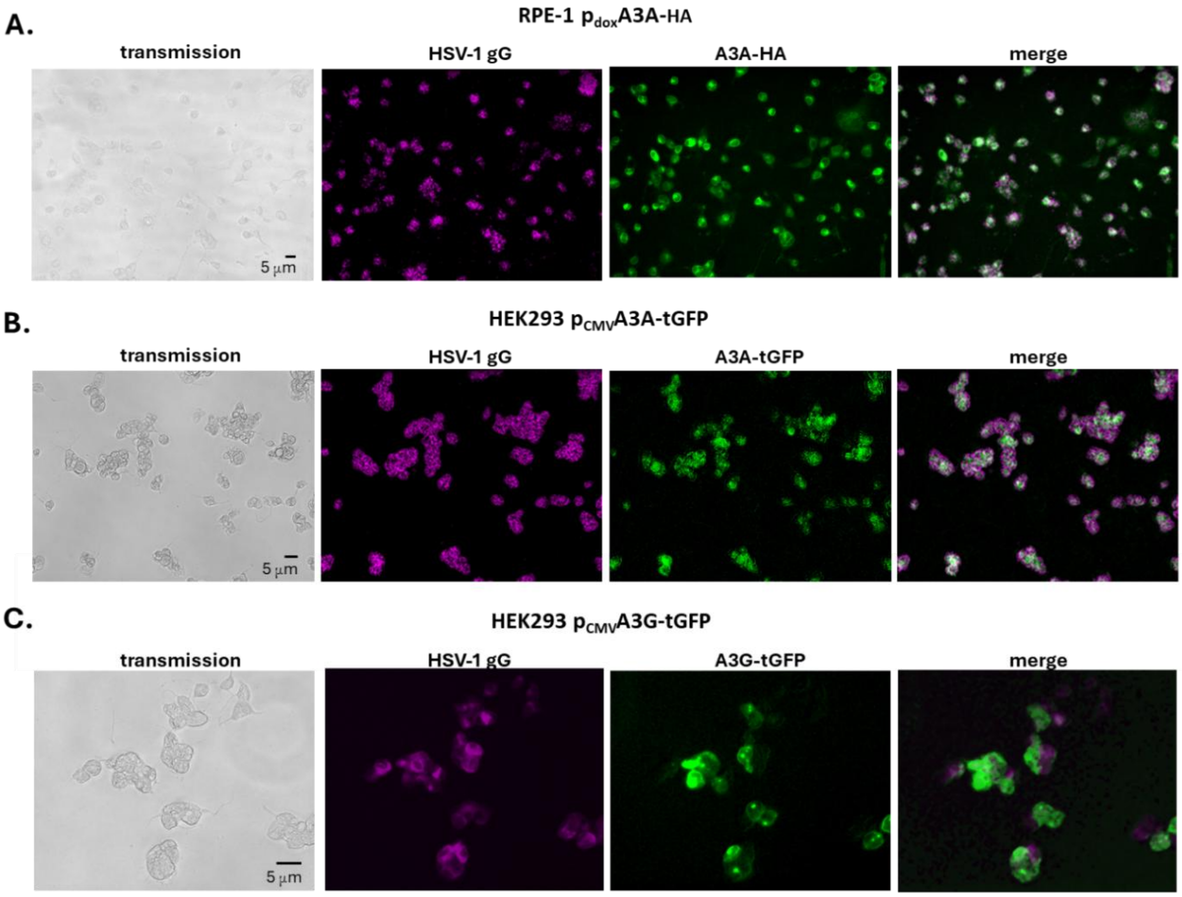

Fig. S6. Immunofluorescent detection of HSV-1 and APOBEC3A or APOBEC3G expression. (A) RPE-1 p_{dox}A3A-HA cells. (B) HEK293 p_{CMV}A3A-tGFP cells. (C) HEK293 p_{CMV}A3G-tGFP cells. HSV-1 was detected using anti-HSV-1 glycoprotein B antibody (magenta). A3A-HA was detected using anti-HA antibody (green), while A3A-tGFP and A3G-tGFP were visualized by turboGFP fluorescence (green).

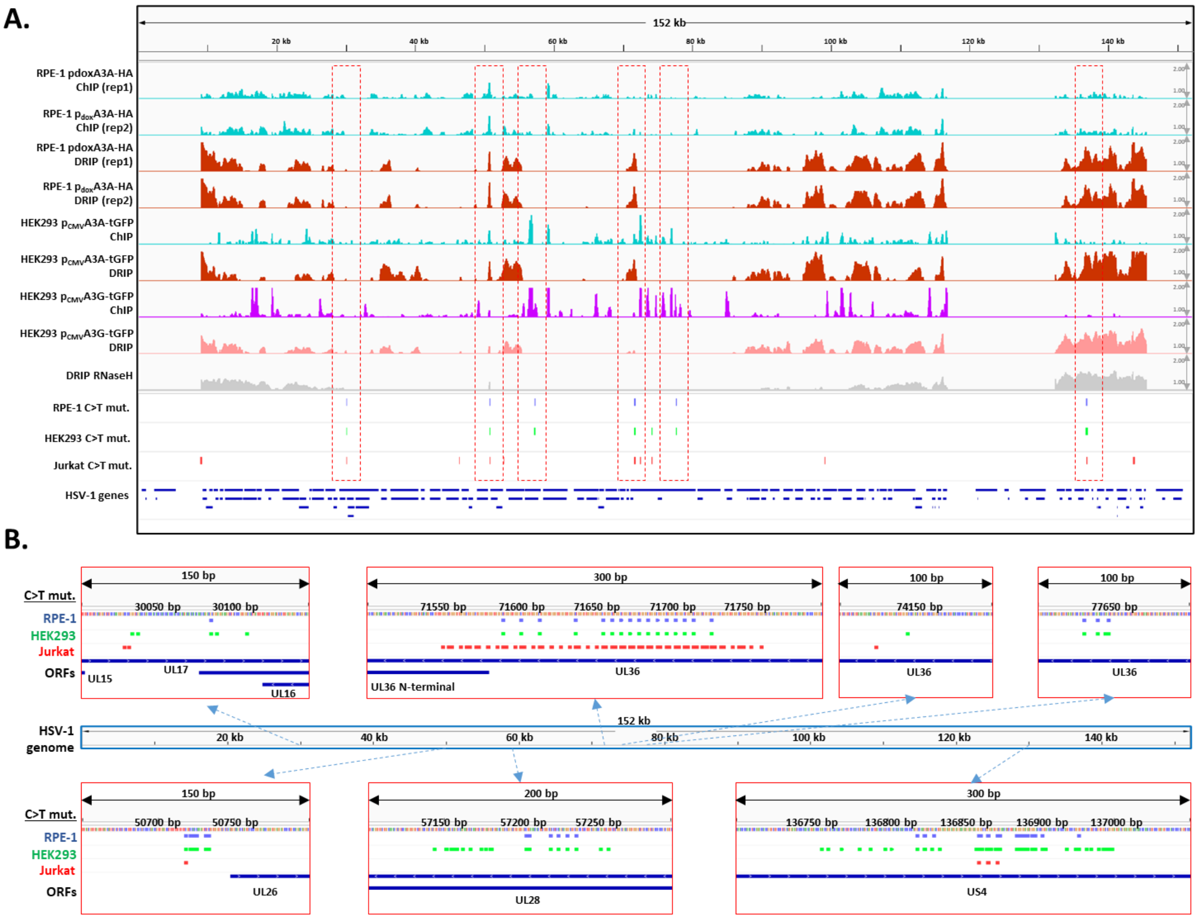

Fig. S7. APOBEC-type C→T mutation hotspots shared between ChIP, DRIP, and input HSV-1 genomes. (A) Genome browser snapshot showing the ChIP and DRIP signals as well as the C>T mutations in RPE-1 p_{dox}A3A-HA, HEK293 p_{CMV}A3A-tGFP, and HEK293 p_{CMV}A3G-tGFP cells. C-to-T mutations are represented by vertical lines. Data was normalized to input. (B) High-resolution genome browser view of C→T mutation hotspots in the HSV-1 genome. Seven regions are highlighted and magnified to display local mutation patterns.

A.

Western blot anti-APOBEC3G (H-63) (Sc-48820, Santa Cruz Biotechnology, Inc)

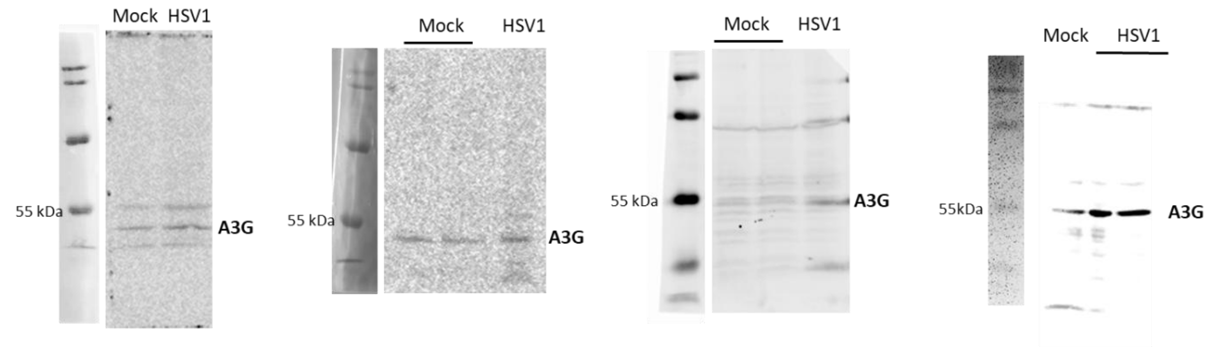

B.

Western blot anti-APOBEC3A (D-23) (Sc-130688, Santa Cruz Biotechnology, Inc)

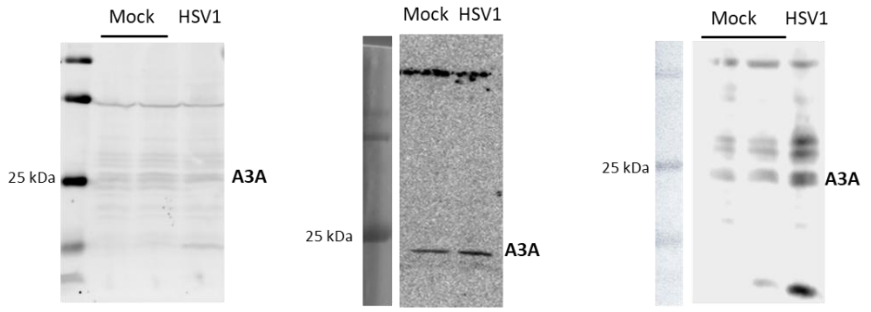

Fig. S8. Western blot analysis of APOBEC3A and APOBEC3G expression. Original images. The antibodies detected a dominant band at the expected molecular weights of A3A (20 kDa) and A3G (40 kDa). Upregulation of A3G and A3A enzymes upon HSV-1 infection is apparent.

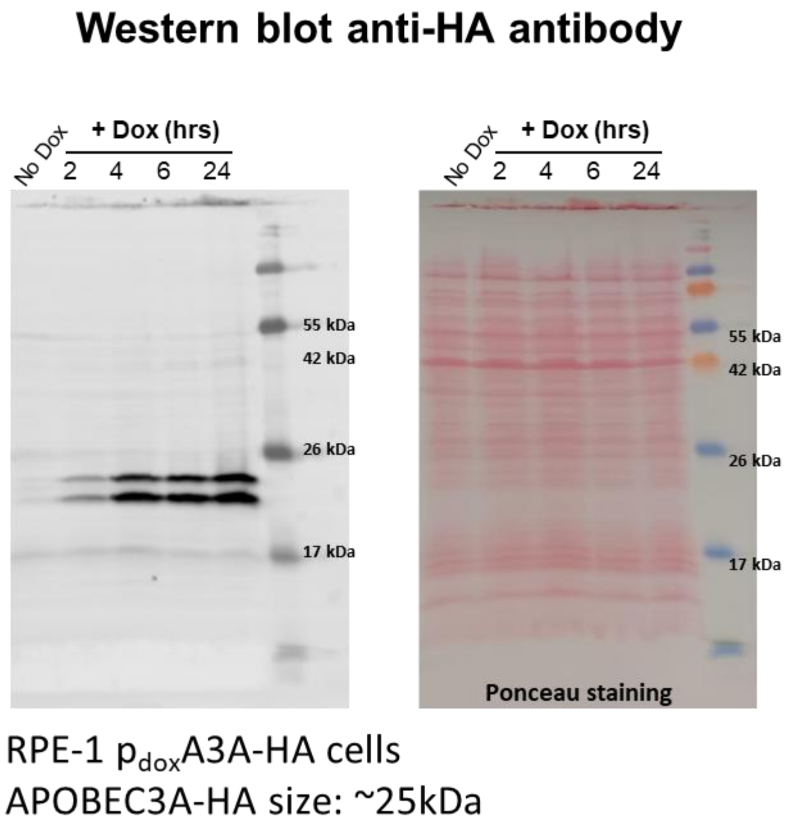

Fig. S9. Western blot analysis of APOBEC3A-HA expression in RPE-1 p_{dox}A3A-HA cells. Original image. The antibody detected a dominant band at the expected molecular weights of A3A-HA (25 kDa) that scaled with the time of doxycycline (dox) induction. The two bands observed in RPE-1 A3A-HA lysates both correspond to A3A-HA. The APOBEC3A transgene contains a second AUG at codon 13, which can serve as an alternative translation start site and produces a slightly smaller N-terminally truncated isoform; in our lentiviral constructs, this shorter isoform is often expressed more prominently, giving rise to the characteristic doublet.

Fig. S10. Amino acid substitutions in HSV-1 proteins arising from APOBEC-mediated mutations. Aligned amino acid sequences of representative HSV-1 protein-coding genes that accumulate APOBEC-type C→T mutations are shown, highlighting the resulting amino acid changes.

UL36

UL36 (WT)
MIAGTPPHSTMERGGDRDIVVTGARNQFAPDLEPGGSVSCMRSSLSFLSLIFDVGPRDVL
UL36 (MUT)
MIAGTPPHSTMERGGDRDIVVTGARNQFAPDLEPGGSVSCMRSSLSFLSLIFDVGPRDVL

UL36 (WT)
SAEAIEGCLVEGGEWTRATAGPGPPRMCSIVELPNFLEYPGARGGLRCVFSRVYGEVGFF
UL36 (MUT)
SAEAIEGCLVEGGEWTRATAGPGPPRMCSIVELPNFLEYPGARGGLRCVFSRVYGEVGFF

UL36 (WT)
GEPAAGLLETQCPAHTFFAGPWALRPLSYTLLTIGPLGMGLFRDGDTAYLFDPHGLPEGT
UL36 (MUT)
GEPAAGLLETQCPAHTFFAGPWALRPLSYTLLTIGPLGMGLFRDGDTAYLFDPHGLPEGT

UL36 (WT)
PAFIAKVRAGDMYPYLTYYTRDRPDVRWAGAMVFFVPSGPEPAAPADLTAAALHLYGASE
UL36 (MUT)
PAFIAKVRAGDMYPYLTYYTRDRPDVRWAGAMVFFVPSGPEPAAPADLTAAALHLYGASE

UL36 (WT)
TYMQDEAFSERRVAITHPLRGEIAGLGEPCVGVGPREGGGGPGPHPPTAAQSPPPTRARR
UL36 (MUT)
TYMQDEAFSERRVAITHPLRGEIAGLGEPCVGVGPREGGGGPGPHPPTAAQSPPPTRARR

UL36 (WT)
DDRASETSRGTAGPSAKPEAKRPNRAPDDVWAVALKGTPPTDPPSADPPSAIPPPPPSAP
UL36 (MUT)
DDRASETSRGTAGPSAKPEAKRPNRAPDDVWAVALKGTPPTDPPSADPPSAIPPPPPSAP

UL36 (WT)
KTPAAEAAEEDDDDMRVLEMGVVPVGRHRARYSAGLPKRRRPTWTPPSSVEDLTSGEKTK
UL36 (MUT)
KTPAAEAAEEDDDDMRVLEMGVVPVGRHRARYSAGLPKRRRPTWTPPSSVEDLTSGEKTK

UL36 (WT)
RSAPPAKTKKKSTPKGKTPVGAAVPASVPEPVLASAPPDPAGPPVAEAGEDDGPMVPASS
UL36 (MUT)
RSAPPAKTKKKSTPKGKTPVGAAVPASVPEPVLASAPPDPAGPPVAEAGEDDGPMVPASS

UL36 (WT)
QALEALKTRRSPEPPGADLAQLFEAHPNVAATAVKFTACSATLAREVAACSRLTISALRS

UL36 (MUT)
QALEALKTRRSPEPPGADLAQLFEAHPNVAATAVKFTACSATLAREVAACSRLTISALRS

UL36 (WT)
PYPASPGLLELCVIFFFERVLAFLIENGARTHTQAGVAGPAAALLEFTLSMLPRKTAVGD
UL36 (MUT)
PYPASPGLLELCVIFFFERVLAFLIENGARTHTQAGVAGPAAALLEFTLSMLPRKTAVGD

UL36 (WT)
FLASTRLSLADVAAHLPLVQHVLDENSLIGRLALAKLILVARDVIRETDAFYGELADLEL
UL36 (MUT)
FLASTRLSLADVAAHLPLVQHVLDENSLIGRLALAKLILVARDVIRETDAFYGELADLEL

UL36 (WT)
QLRAAPPANLYTRLGEWLLERSQAHPDTLFAPATPTHPEPLLYRVQALAKFARGEEIRVE
UL36 (MUT)
QLRAAPPANLYTRLGEWLLERSQAHPDTLFAPATPTHPEPLLYRVQALAKFARGEEIRVE

UL36 (WT)
AEDRQMREALDALARGVDAVSQHAGPLGVMPAPAGAAPQGAPRPPPLGPEAVQVRLEEVR
UL36 (MUT)
AEDRQMREALDALARGVDAVSQHAGPLGVMPAPAGAAPQGAPRPPPLGPEAVQVRLEEVR

UL36 (WT)
TQARRAIEGAVKEYFYRGAVYSAKALQASDNNDRRFHVASAAVVPVVQLLESLPVFDQHT
UL36 (MUT)
TQARRAIEGAVKEYFYRGAVYSAKALQASDNNDRRFHVASAAVVPVVQLLESLPVFDQHT

UL36 (WT)
RDIAQRAAIPAPPPIATSPTAILLRDLIQRGQTLDAPEDLAAWLSVLTDAANQGLIERKP
UL36 (MUT)
RDIAQRAAIPAPPPIATSPTAILLRDLIQRGQTLDAPEDLAAWLSVLTDAANQGLIERKP

UL36 (WT)
LDELARSIRDINDQQARRSSGLAELRRFDALDAALGQQLDSDAAFVPAPGASPYPDDGGL
UL36 (MUT)
LDELARSIRDINDQQARRSSGLAELRRFDALDAALGQQLDSDAAFVPAPGASPYPDDGGL

UL36 (WT)
SPEATRMAEEALRQARAMDAAKLTAELAPDARARLRERARSLEAMLEGARERAKVARDAR
UL36 (MUT)
SPEATRMAEEALRQARAMDAAKLTAELAPDARARLRERARSLEAMLEGARERAKVARDAR

UL36 (WT)
EKFLHKLQGVLRPLPDFVGLKACPAVLATLRASLPAGWSDLPEAVRGAPPEVTAALRADM

UL36 (MUT)
EKFLHKLQGVLRPLPDFVGLKACPAVLATLRASLPAGWSDLPEAVRGAPPEVTAALRADM

UL36 (WT)
WGLLGQYRDALEHPTPDTATALSGLHPSFVVVLKNLFADAPETPFLLQFFADHAPIIAHA
UL36 (MUT)
WGLLGQYRDALEHPTPDTATALSGLHPSFVVVLKNLFADAPETPFLLQFFADHAPIIAHA

UL36 (WT)
VSNAINAGSAAVATADPASTVDAAVRAHRVLVDAVTALGAAASDPASPLAFLAAMADSAA
UL36 (MUT)
VSNAINAGSAAVATADPASTVDAAVRAHRVLVDAVTALGAAASDPASPLAFLAAMADSAA

UL36 (WT)
GYVKATRLALDARGAIAQLTTLGSAAADLVVQVRRAANQPEGEHASLIQAATRATTGARE
UL36 (MUT)
GYVKATRLALDARGAIAQLTTLGSAAADLVVQVRRAANQPEGEHASLIQAATRATTGARE

UL36 (WT)
SLAGHEGRFGGLLHAEGTAGDHSPSGRALQELGKVIGATRRRADELEAAIADLREKMAAQ
UL36 (MUT)
SLAGHEGRFGGLLHAEGTAGDHSPSGRALQELGKVIGATRRRADELEAAIADLREKMAAQ

UL36 (WT)
RARSSHERWAADVEAVLDRVESGAEFDVVELRRLQALAGTHGYNPRDFRKRAEQALGTNA
UL36 (MUT)
RARSSHERWAADVEAVLDRVESGAEFDVVELRRLQALAGTHGYNPRDFRKRAEQALGTNA

UL36 (WT)
KAVTLALETALAFNPYTPENQRHPMLPPLAAIHRIDWSAAFGAAADTYADMFRVDTEPLA
UL36 (MUT)
KAVTLALETALAFNPYTPENQRHPMLPPLAAIHRIDWSAAFGAAADTYADMFRVDTEPLA

UL36 (WT)
RLLRLAGGLLERAQANDGFIDYHEAVLHLSEDLGGVPALRQYVPFFQKGYAEYVDIRDRL
UL36 (MUT)
RLLRLAGGLLERAQANDGFIDYHEAVLHLSEDLGGVPALRQYVPFFQKGYAEYVDIRDRL

UL36 (WT)
DALRADARRAIGSVALDLAAAAEEISAVRNDPAAAAELVRAGVTLPCPSEDALVACVAAL
UL36 (MUT)
DALRADARRAIGSVALDLAAAAEEISAVRNDPAAAAELVRAGVTLPCPSEDALVACVAAL

UL36 (WT)
ERVDQSPVKDTAYADYVAFVTRQDLADTKDAVVRAKQQRAEATERVTAGLREVLAARERR
UL36 (MUT)
ERVDQSPVKDTAYADYVAFVTRQDLADTKDAVVRAKQQRAEATERVTAGLREVLAARERR

UL36 (WT)
AQLEAEGLANLKTLLKVVAVPATVAKTLDQARSAEEIADQVEILLDQTEKARELDVQAVA
UL36 (MUT)
AQLEAEGLANLKTLLKVVAVPATVAKTLDQARSAEEIADQVEILLDQTEKARELDVQAVA

UL36 (WT)
WLEHAQRTFETHPLNAASGDGPGLLTRQGARLQALFDTRRRVEALRRSLEEAEAEWDEVW
UL36 (MUT)
WLEHAQRTFETHPLNAASGDGPGLLTRQGARLQALFDTRRRVEALRRSLEEAEAEWDEVW

UL36 (WT)
GRFGRVRGGAWKSPEGFRAACEQLRALQDTTNTVSGLRAQRDYERLPAKYQGVLGAKSAE
UL36 (MUT)
GRFGRVRGGAWKSPEGFRAACEQLRALQDTTNTVSGLRAQRDYERLPAKYQGVLGAKSAE

UL36 (WT)
RAGAVEELGGRVAQHADLSARLRDEVVPRVAWEMNFDTLGGLLAEFDAVAGDLAPWAVEE
UL36 (MUT)
RAGAVEELGGRVAQHADLSARLRDEVVPRVAWEMNFDTLGGLLAEFDAVAGDLAPWAVEE

UL36 (WT)
FRGARELIQRRMGLYSAYAKATGQTGAGAAAAPAPLLVDLRALDARARASAPPGQEADPQ
UL36 (MUT)
FRGARELIQRRMGLYSAYAKATGQTGAGAAAAPAPLLVDLRALDARARASAPPGQEADPQ

UL36 (WT)
MLRRRGEAYLRVSGGPGPLVLREATSTLDRPFAPSFLVPDGTPLQYALCFPAVTDKLGAL
UL36 (MUT)
MLRRRGEAYLRVSGGPGPLVLREATSTLDRPFAPSFLVPDGTPLQYALCFPAVTDKLGAL

UL36 (WT)
LMCPEAACIRPPLPTDTLESASTVTAMYVITVINRLQLALSDAQAANFQLFGRFVRHRQA
UL36 (MUT)
LMCPEAACIRPPLPTDTLESASTVTAMYVITVINRLQLALSDAQAANFQLFGRFVRHRQA

UL36 (WT)
RWGASMDAAAELYVALVATTLTREFGCRWAQLEWGGDAAAPGPPLGPHSSTRHRVSFNEN
UL36 (MUT)
RWGASMDAAAELYVALVATTLTREFGCRWAQLEWGGDAAAPGPPLGPHSSTRHCVSFNEN

UL36 (WT)
DVLVALVASSPEHIYTFWRLDLVRQHEYMHLTLPRAFQNAADSMLFVQRLTPHPDARIRV
UL36 (MUT)
DVLVALVASSPEHIYTFWRLDLVRQHEYMHLTLPRAFQNAADSMLFVQRLTPHPDARIRV

UL36 (WT)
LPVFSTGGPPTRGLMFGTRLADWRRGKLSETDPLAPWRSVPELGTERGAALGKLSPAQAL

UL36 (MUT)
LPVFSTGGPPTRGLMFGTRLADWRRGKLSETDPLAPWRSVPELGTERGAALGKLSPAQAL

____

UL36 (WT)
AAVSVLGRMCLPSTALAALWTCMFPDDYTEYDSFDALLTARLESGQTLSPSGGREASPPA
UL36 (MUT)
AAVSVLGRMCLPSTALAALWTCMFPDDYTEYDSFDALLTARLESGQTLSPSGGREASPPA

____

UL36 (WT)
PPNALYRPTGQHVAVPAAATHRTPAARVTAMDLVLAAVLLGAPVVVALRNTTAFSRESEL
UL36 (MUT)
PPNALYRPTGQHVAVPAAATHRTPAARVTAMDLVLAAVLLGAPVVVALRNTTAFSRESEL

____

UL36 (WT)
ELCLTLFDSRARGPDAALRDAVSSDIETWAVRLLHADLNPIENACLAAQLPRLSALIAER
UL36 (MUT)
ELCLTLFDSRARGPDAALRDAVSSDIETWAVRLLHADLNPIENACLAAQLPRLSALIAER

____

UL36 (WT)
PLARGPPCLVLVDISMTPVAVLWENPDPPGPPDVRFVGSEATEELPFVAGGEDVLAASAT
UL36 (MUT)
PLARGPPCLVLVDISMTPVAVLWENPDPPGPPDVRVVGSEATEELPFVAGGEDGLAARAT

___________________________________.________________ ___ __

UL36 (WT)
DEDPFLARAILGRPFDASLLSGELFPGHPVYQRAPDDQSPSVPNPTPGPADLVGTEGSLG
UL36 (MUT)
DEDPLLARAILGRPFDASLLSGELFPGHPVYQRAPDDQSPSVPNPTPGPADLVGTEGSLG

____:____

UL36 (WT)
PGSLAPTLFTDATPGEPVPPRMWAWIHGLEELASDDSGGPAPLLAPDPLSPTADQSVPTS
UL36 (MUT)
PGSLAPTLFTDATPGEPVPPRMWAWIHGLEELASDDSGGPAPLLAPDPLSPPADQSVPTS

____.________

UL36 (WT)
QCAPRPPGPAVTAREARPGVPAESTRPAPVGPRDDFRRLPSPQSSPAPPDATAPRPPASS
UL36 (MUT)
QCAPRPPGPAVTAREARPGVPAESTRPAPVGPRDDFRRLPSPQSSPAPPDATAPPPPPPP

____ __...

UL36 (WT)
RASAASSSGSRARRHRRARSLARATQASATTQGWRPPALPDTVAPVTDFARPPAPPKPPE
UL36 (MUT)
RASGTVSSGSRARRHRRARSLARATQASATTQGWRPPALPDTVAPVTDFARPPAPPKPPE

___.: ____

UL36 (WT)
PALHALVSGVPLPLGPQFAGQASPALPIDPVPPPVATGTVLPGGENRRPPLTSGPAPTPP
UL36 (MUT)
PALHALVSGVPLPLGPQFAGQASPALPIDPVPPPVATGTVLPGGENRRPPLTSGPAPTPP

____

UL36 (WT)
RVPVGGPQRRLTRPAVASLSESRESLPSPWDPADPTAPVLGRNPAEPTSSSPAGPSPPPP
UL36 (MUT)
RVPVGGPQRRLTRPAVASLSESRESLPSPWDPADPTAPVLGRNPAEPTSSSPAGPSPPPP

____

UL36 (WT)
AVQPVTPPPTSGPPPTYLTLEGGVTPGGPVSRRPTTRQPVATPTTSARPRGHLTVNRLSA
UL36 (MUT)
AVQPVTPPPTSGPPPTYLTLEGGVTPGGPVSRRPTTRQPVATPTTSARPRGHLTVNRLSS
____:

UL36 (WT)
PQPQPQPQPQPQPQPQPQPQPQPQPQPQPQPQPQPQPQPQPQPQPQPQPQPQPQPQPQPQ
UL36 (MUT) PPPSP*P*P*P*P*P*P*P*P*P*P*P*P*P*P*P*P*P*P*P*P*P*P*P*P*P*P*P*
_:_:_* *

UL36 (WT)
PQPQPQPQPQPQNGHVAPGEYPAVRFRAPQNRPSVPASASSTNPRTGSSLSGVSSWASSL
UL36 (MUT)
P*P*P*P*P*P*NGHVAPGEYPAVRFRAPQNRPSVPASASSTNPRTGSSLSGVSSWASSL
* * * * * *

UL36 (WT)
ALHIDATPPPVSLLQTLYVSDDEDSDTTSLFLSDSEAEALDPLPREPHSPITNEPFSALS
UL36 (MUT)
ALHIDATPPPVSLLQTLYVSDDEDSDTTSLFLSDSEAEALDPLPREPHSPITNEPFSALS

UL36 (WT)
ADDSQEVTRLQFGPPPVSANAVLSRRYVQRTGRSALAVLIRACYRLQQQLQRTRRALLHH
UL36 (MUT)
ADDSQEVTRLQFGPPPVSANAVLSRRYVQRTGRSALAVLIRACYRLQQQLQRTRRALLHH

UL36 (WT) SDAVLTSLHHVRMLLG
UL36 (MUT) SDAVLTSLHHVRMLLG

UL15

UL15 (WT)
MFGQQLASDVQQYLERLEKQRQLKVGADEASAGLTMGGDALRVPFLDFATATPKRHQTVV
UL15 (MUT)
MFGQQLASDVQQYLERLEKQRQLKVGADEASAGLTMGGDALRVPFLDFATATPKRHQTVV

____

UL15 (WT)
PGVGTLHDCCEHSPLFSAVARRLLFNSLVPAQLKGRDFGGDHTAKLEFLAPELVRAVARL
UL15 (MUT)
PGVGTLHDCCEHSPLFSAVARRLLFNSLVPAQLKGRDFGGDHTAKLEFLAPELVRAVARL

____

UL15 (WT)
RFKECAPADVVPQRNAYYSVLNTFQALHRSEAFRQLVHFVRDFAQLLKTSFRASSLTETT

UL15 (MUT)
RFKECAPADVVPQRNAYYSVLNTFQALHRSEAFRQLVHFVRDFAQLLKTSFRASSLTETT

UL15 (WT)
GPPKKRAKVDVATHGRTYGTLELFQKMILMHATYFLAAVLLGDHAEQVNTFLRLVFEIPL
UL15 (MUT)
GPPKKRAKVDVATHGRTYGTLELFQKMILMHATYFLAAVLLGDHAEQVNTFLRLVFEIPL

UL15 (WT)
FSDAAVRHFRQRATVFLVPRRHGKTWFLVPLIALSLASFRGIKIGYTAHIRKATEPVFEE
UL15 (MUT)
FSDAAVRHFRQRATVFLVPRRHGKTWFLVPLIALSLASFRGIKIGYTAHIRKATEPVFEE

UL15 (WT)
IDACLRGWFGSARVDHVKGETISFSFPDGSRSTIVFASSHNTNVSPLFFRMALPRGPGST
UL15 (MUT)
IDACLRGWFGSARVDHVKGETISFSFPDGSRSTIVFASSHNTNVSPLFFGMALPRGPVST

UL15 (WT)
RPTPTHPHTHTTRRGRKVCPVGTDFYSGSLEEARATARATVGQLVANRRLMYEEKTHRRH
UL15 (MUT)
PHTHTHTHTHPPTRGRKVCPVGTDFYSGSLEEARATARATVGQLVANRRLMYEEKTHRRH

UL15 (WT)
PALVGGMLSAPHRPPTTSCRRSVMQGRRGACSRVTVSTGWPKRSGHRLASPWTARVHWAI
UL15 (MUT)
PALVGGMLSAPHRPPTTSCRRSVMQGRRGACSRVTVSTGWPKRSGHRLASPWTARVHWAI

UL15 (WT)
LAHRHGATQGQDSVTTEESHSALAESMWGGGAQDSQPPGWLVILARSHPRWRALLPGGQS
UL15 (MUT)
LAHRHGATQGQDSVTTEESHSALAESMWGGGAQDSQPPGWLVILARSHPRWRALLPGGQS

UL15 (WT) DPRSLSMAQQ
UL15 (MUT) DPRSLSMAQQ

UL46

UL46 (WT)
MQRRTRGASSLRLARCLTPANLIRGDNAGVPERRIFGGCLLPTPEGLLSAAVGALRQRSD
UL46 (MUT)
MQRRTRGASSLRLARCLTPANLIRGDNAGVPERRIFGGCLLPTPEGLLSAAVGALRQRSD

UL46 (WT)
DAQPAFLTCTDRSVRLAARQHNTVPESLIVDGLASDPHYEYIRHYASAATQALGEVELPG
UL46 (MUT)
DAQPAFLTCTDRSVRLAARQHNTVPESLIVDGLASDPHYEYIRHYASAATQALGEVELPG

UL46 (WT)
GQLSRAILTQYWKYLQTVVPSGLDVPEDPVGDCDPSLHVLLRPTLAPKLLARTPFKSGAA
UL46 (MUT)
GQLSRAILTQYWKYLQTVVPSGLDVPEDPVGDCDPSLHVLLRPTLAPKLLARTPFKSGAA

UL46 (WT)
AAKYAATVAGLRDALHRIQQYMFFMRPADPSRPSTDTALRLNELLAYVSVLYRWASWMLW
UL46 (MUT)
AAKYAATVAGLRDALHRIQQYMFFMRPADPSRPSTDTALRLNELLAYVSVLYRWASWMLW

UL46 (WT)
TTDKHVCHRLSPSNRRFLPLGGSPEAPAETFARHLDRGPSGTTGSMQCMALRAAVSDVLG
UL46 (MUT)
TTDKHVCHRLSPSNRRFLPLGGSPEAPAETFARHLDRGPSGTTGSMQCMALRAAVSDVLG

UL46 (WT)
HLTCLANLWQTGKRSGGTYGTVDTVVSTVEVLSIVHHHAQYIINATLTGYGVWATDSLNN
UL46 (MUT)
HLTCLANLWQTGKRSGGTYGTVDTVVSTVEVLSIVHHHAQYIINATLTGYGVWATDSLNN

UL46 (WT)
EYLRAAVDSQERFCRTTAPLFPTMTAPSWARMELSIKAWFGAALAADLLRSGAPSLHYES
UL46 (MUT)
EYLRAAVDSQERFCRTTAPLFPTMTAPSWARMELSIKAWFGAALAADLLRSGAPSLHYES

UL46 (WT)
ILRLVASRRTTWSAGPPPDDMARGPGGHRAGGGTCREKIQRARRDNEPPPLPRPRLHSTP
UL46 (MUT)
ILRLVASRRTTWSAGPPPDDMARGPGGHRAGGGTCREKIQRARRDNEPPPLPRPRLHSTP

UL46 (WT)
APSTRRFRRRRADGAGPPLPDADDPVAEPPAAAAQPATYYTHMGEVPPRLPARNVAGPDR
UL46 (MUT)
APSTRRFRRRRADGAGPPLPDADDPVAEPPAAAAQPATYYTHMGEVPPRLPARNVAGPDR

UL46 (WT)
RPPAATCPLLVRRASLGSLDRPRVWGPAPEGEPDQMEATYLTADDDDDDARRKATHAASA
UL46 (MUT)
RPPAATCPLLVRRASLGSLDRPRVWGPAPEGEPDQMEATYLTRDDDDDDGHGKAFHPAGA
____ _______.. __ _._._

UL46 (WT)
RERHAPYEDDESIYETVSEDGGRVYEEIPWMRVYENVCVNTANAAPASPYIEAENPLYDW
UL46 (MUT)
DERHAPYEDDESIYETVSEDGGRVYEEIPWMRVYENVCVNTANAAPASPYIEAENPLYDW

UL46 (WT)
GGSALFSPPGRTGPPPPPLSPSPVLARHRANALTNDGPTNVAALSALLTKLKREGRRSR
UL46 (MUT)
GGSALFSPPGRTGPPPPPLSPSPVLARHRANALTNDGPTNVAALSALLTKLKREGRRSR

US4

US4 (WT)
MSPGAMRAVVPIIPFLLVLVGVSGVPTNVSSTTQPQLQTTGRPSHEAPNMTQTGTTDSPT
US4 (MUT
MSPGAMRAVVPIIPFLLVLVGVSGVPTNVSSTTQPQLQTTGRPSHEAPNMTQTGTTDSPT

US4 (WT)
AISLTTPDHTPPMPSIGLEEEEEEEEEGAGDGEHLKGGDGTRDTLPQSPGPAVPLAGDDEK
US4 (MUT)
AISLTTPDHTPPMPSIGEEEEEEEEEEEEEEEEEKGGDGTRDTLPQSPGPAVPLAGDDEK
___________________ _________ :_ _____________________________

US4 (WT)
DKPNRPVVPPPGPNNSPARPETSRPKTPPTSIGPLATRPTTQLPSKGRPLVPTPQHTPLF
US4 (MUT)
DKPNRPVVPPPGPNNSPARPETSRPKTPPTSIGPLATRPTTQLPSKGRPLVPTPQHTPLF

US4 (WT)
SFLTASPALDTLFVVSTVIHTLSFVCIVAMATHLCGGWSRRGRRTHPSVRYVCLPPERG
US4 (MUT)
SFLTASPALDTLFVVSTVIHTLSFVCIVAMATHLCGGWSRRGRRTHPSVRYVCLPPERG

Table S1: APOBEC mutational hotspots identified in RPE-1 pdoxA3A-HA, HEK293-AD pCMVA3A-tGFP, and HEK293-AD pCMVA3G-tGFP cells.

A. **RPE-1 p_{dox}A3A-HA hotspots**

Hotspots	# C>T	input	A3A-HA ChIP (rep1)	A3A-HA ChIP (rep2)	DRIP (rep1)	DRIP (rep2)
1. 30085-30087	1			+		
2. 50718-50735	7	+	+	+	+	+
3. 57204-57239	6		+	+		
4. 71589-71729	16	+	+	+	+	+
5. 77630-77648	3	+		+		
6. 136819-136928	19		+	+		
total:	52	24	20	38	10	10

B. **HEK293 p_{CMV}A3A-tGFP hotspots**

Hotspots	# C>T	input	A3A-tGFP ChIP	DRIP
1. 30033-30091	4		+	
2. 50718-50735	7	+	+	+
3. 57143-57260	19		+	
4. 71589-71729	16	+	+	+
5. 74144-74146	1		+	
6. 77630-77648	3	+		+
7. 136756-136950	46		+	+
total:	96	23	80	13

C. **HEK293 p_{CMV}A3G-tGFP hotspots**

Hotspots	# C>T	input	A3G-tGFP ChIP	DRIP
1. 30085-30087	3		+	
2. 50718-50735	7	+	+	+
3. 57204-57239	7		+	
4. 71589-71729	14	+	+	+
5. 77630-77648	4	+	+	+
6. 136819-136928	28	+	+	
total:	63	25	48	11

(A) RPE-1 pdoxA3A-HA hotspots. Mutational hotspots identified in doxycycline-inducible A3A-HA expressing RPE-1 cells. Each row represents a hotspot region with coordinates indicating HSV-1 genome positions. The #C→T column shows the number of C→T mutations within each hotspot. Plus signs (+) indicate enrichment above background in input DNA, A3A-HA ChIP-seq (two biological replicates), and DRIP-seq (two biological replicates). The total row shows cumulative values across all hotspots for each dataset (n = total mutations or number of hotspots with enrichment). (B) HEK293 pCMVA3A-tGFP hotspots. Mutational hotspots identified in HEK293-AD cells constitutively expressing A3A-tGFP. Table structure as in panel A. (C) HEK293 pCMVA3G-tGFP hotspots.

Mutational hotspots identified in HEK293-AD cells constitutively expressing A3G-tGFP. Table structure as in panels A and B.

Table S2: List of qPCR primers used in this study.

Oligo name	sequence
HSV-1_1_F	ATTGCCGTTGGGGTTTTGTG
HSV-1_1_R	CGTAACGCCAATCAAGATCGTC
HSV-1_2_F	AGGCGGTTGACGGTCAAATG
HSV-1_2_R	CCCACATACTTGACGCTGGAG
HSV-1_3_F	TTACGTGGCTGGGTTTTTGG
HSV-1_3_R	TTAATCGGGAGTGGGTTATCCG
HSV-1_4_F	TGTCACGGGAAAGGAAAGAGG
HSV-1_4_R	ACGCCTTTTGTGTGTGTGTG
HSV-1_5_F	TGCGTCTCTGGGTGTTTTTG
HSV-1_5_R	CCCGGGCATGTATTTAACTGC
HSV-1_6_F	AACGCACCAAACAGATGCAG
HSV-1_6_R	TGTTGATGGCCTGTGCTTTC
ZNF554_FW	CGGGGAAAAGCCCTATAAAT
ZNF554_REV	TCCACATTCACTGCATTCGT
