# Supplementary material for: Herpes simplex virus type 1 R-loops are targets for APOBEC-mediated mutagenesis
Source: Genome Biol. 2026 Apr 14;27:169. doi: 10.1186/s13059-026-04078-y (PMC13185267; doi:10.1186/s13059-026-04078-y)
Supplement: Supplementary file 3 — Additional file 3: Supplementary text about the limitations of the study. [file 13059_2026_4078_MOESM3_ESM.pdf]

Additional file 3 - supplementary text

Herpes simplex virus type 1 R-loops are targets for APOBEC-mediated mutagenesis

Márton Miskei, Dóra Varga, Lilla Hornyák, Éva Sipos, Éva Nagy, Qiuzhen Li, Zsolt Karányi, Zoltán Szabó, Rachel DeWeerd, Abby M Green, Dávid Szüts, Eszter Csoma, Lóránt Székvölgyi

Our study has the following limitations:

1) **Experimental Conditions:** The experimental conditions were optimized for maximal cell viability (minimal cell death) and a high virus burden after the infection with HSV-1. This design aimed to detect the activity of host APOBEC3 enzymes on invading viruses and identify mutational signatures that inactivate viral genes. Consequently, we did not investigate shedding viruses released in the later stages of infection from cytopathic host cells, as deleterious APOBEC mutations are likely removed by selection for functional virions. Mature viruses are expected to undergo strong positive selection for wild-type alleles (or beneficial mutations), while harmful mutations are negatively selected and remain undetectable in the shedding virus population.

2) **Relocalization pattern of A3G and A3A upon HSV-1 infection:** It is well established that APOBEC3G is primarily localized in the cytoplasm, while APOBEC3A is predominantly distributed in the nucleus under normal conditions [1]. Moreover, herpesviruses, including HSV-1, are known to manipulate the localization and activity of APOBEC3 proteins to evade host immune defenses [2,3]. While HSV-1 initially upregulates APOBEC3 expression as part of the host immune response, it may also employ mechanisms to counteract the antiviral effects of A3A and A3G proteins. This dynamic interplay could influence the localization patterns of APOBEC3 proteins during infection. Under our experimental conditions, however, we could not definitively determine whether the observed A3G and A3A foci are nuclear or cytoplasmic, and we have therefore refrained from making specific claims about their localization. This limitation arises from the use of a cell lysis buffer that disrupts nuclear membrane integrity, as the labeling conditions were specifically optimized to quantify APOBEC protein expression levels and RNA:DNA hybrid levels using laser scanning cytometry (LSC).

3) **Interpretation of A3A and A3G ChIP peak overlap:** In Figure 1D, ChIP-seq peaks for A3A and A3G show substantial overlap despite the fact that these enzymes recognize partially distinct DNA sequence motifs at the single-nucleotide level. We interpret this primarily as a consequence of shared structural rather than strictly sequence-based requirements for substrate engagement. A3A and A3G

differ in oligomeric state and DNA-binding properties (A3A is monomeric with cooperative dimerization upon substrate binding, whereas A3G is predominantly dimeric with minor tetrameric/monomeric species), but both enzymes preferentially act on exposed single-stranded DNA. R-loops generate extended ssDNA within and around RNA:DNA hybrids, creating a permissive structural environment that can be accessed by multiple APOBEC3 family members irrespective of fine sequence preference. Under these conditions, broad ChIP peaks—which represent population-averaged binding across many cells and many adjacent nucleotides—are expected to converge over the same R-loop–associated regions even if the exact cytidines deaminated by A3A and A3G differ. Thus, we interpret the observed A3A/A3G ChIP peak overlap as reflecting a shared structural requirement—accessible ssDNA at or near R-loops—rather than identical sequence specificity, and we view this as consistent with our model of R-loop–guided APOBEC3 recruitment.

4) **Bulk Sequencing:** Our DRIP-seq and ChIP-seq data highlight the average enrichment of R-loop structures and A3A/A3G enzymes across a cell population. DRIP and ChIP enrichments should be interpreted as a statistical ensemble of colocalizations, indicating that when A3A/A3G binding sites statistically tend to associate with R-loops, APOBEC mutagenesis is likely to occur. We lack causative relationships between these encounters and the sequence of biochemical events - regarding R-loop formation, APOBEC enzyme binding, and mutagenesis. To establish these relationships, single-cell sequencing and single-cell cloning of individual HSV-1 DNA molecules would be required to identify R-loops, A3A/A3G enzyme binding sites, and APOBEC mutation signatures per single HSV-1 genome.

5) **ChIP enrichment versus population-wide mutational burden:** ChIP-seq inherently enriches for the subset of HSV-1 genomes that are physically bound by APOBEC3 enzymes, which can accentuate APOBEC-type mutations relative to bulk viral DNA. However, deep whole-viral-DNA sequencing of input samples showed that APOBEC-type C→T/G→A mutations were detectable and statistically overrepresented in these high-coverage input samples relative to other substitution types, and they clustered in the same hotspot regions as in the ChIP datasets, indicating a non-random, population-wide APOBEC imprint rather than a ChIP-specific artifact. In RPE-1 pdoxA3A-HA cells, ChIP, DRIP, RNaseH, and input all revealed the same hotspots with comparable C→T/G→A enrichment. In HEK293-AD A3A-tGFP and A3G-tGFP cells, input samples also showed significant C→T/G→A enrichment, although absolute counts were lower than in ChIP DNA. A small set of hotspots (e.g., 30,033–30,111; 57,143–57,260; 136,756–136,950) appeared only in ChIP samples, consistent with rarer, heavily edited genomes. Overall, these data support a heterogeneous, hotspot-based mutational burden that is broadly detectable in the viral population and further amplified in A3-bound genomes captured by ChIP.

6) **Mechanistic Evidence**: We lack mechanistic evidence for the production of mutant HSV-1 proteins resulting from APOBEC mutagenesis. While we have strong evidence that APOBEC mutagenesis exists in the HSV-1 genome due to host A3A/A3G activity, we could not ascertain the exact proportion of affected virus genomes or isolate and characterize the mutant HSV-1 proteins. However, given the high number of HSV-1 genome equivalents at an MOI of 5, even a small fraction (e.g., 0.0001%) could produce a biologically significant portion of mutant proteins inactivated by host APOBEC3 enzymes. These limitations add to the complexity of studying R-loop mutagenesis in the context of HSV-1 infection and underscore the need for further experiments to fully elucidate these mechanisms.

7) **Contribution of endogenous APOBEC family members to HSV-1 mutagenesis:** We did not determine to what extent the observed HSV-1 mutations reflect the combined activity of endogenous APOBEC3 family members. As an initial attempt to address this in a true APOBEC-null background, we tested avian DT40 cells engineered for inducible human A3A expression; however, because DT40 cells did not support productive HSV-1 infection, this approach could not be pursued further. Clarifying the contribution of endogenous APOBEC enzymes will require dedicated loss-of-function studies and remains an important open question.

8) **Biological significance of APOBEC3-mediated C→T mutations in HSV-1 pathogenesis**: The functional consequences of the APOBEC3-mediated C→T mutations identified in the HSV-1 genome remain unresolved. Our primary objective was to establish a mechanistic link between viral R-loop formation and APOBEC3-driven mutagenesis. HSV-1 served as a suitable model because it forms R-loops during replication and/or transcription and is permissive to APOBEC3 activity under our conditions, enabling proof-of-principle that viral R-loops can act as substrates for APOBEC3 enzymes. Whether the resulting mutations ultimately confer antiviral protection, drive viral evolution, or have other roles in HSV-1 biology remains to be determined. Jurkat cells express detectable APOBEC3G, yet this level is insufficient to restrict Vif-deficient HIV-1 [4], whereas we observe clear APOBEC-type hotspots in the HSV-1 genome in Jurkat and APOBEC3 overexpression systems. This likely reflects differences in viral life cycles and APOBEC3 engagement: HIV-1 restriction requires efficient packaging of APOBEC3G into virions, whereas HSV-1 replicates its large double-stranded DNA genome in the nucleus, where transcription- and replication-associated R-loops expose single-stranded DNA to nuclear APOBEC3A/APOBEC3G. Under these conditions, even moderate APOBEC3 activity can leave a detectable, R-loop–associated mutational imprint without global viral collapse. Overall, our data support a model in which APOBEC3 enzymes impose a non-uniform, hotspot-based mutational burden on HSV-1 rather than uniform genome-wide hypermutation. Clarifying the net impact of this editing on HSV-1 fitness and pathogenesis will require in vivo models and longitudinal evolution experiments combining defined APOBEC3 genotypes with deep viral sequencing and functional analysis of recurrent mutations in key viral genes.
